# Observation of nonlinear fractal higher order topological insulator

**DOI:** 10.1038/s41377-024-01611-1

**Published:** 2024-09-20

**Authors:** Hua Zhong, Victor O. Kompanets, Yiqi Zhang, Yaroslav V. Kartashov, Meng Cao, Yongdong Li, Sergei A. Zhuravitskii, Nikolay N. Skryabin, Ivan V. Dyakonov, Alexander A. Kalinkin, Sergei P. Kulik, Sergey V. Chekalin, Victor N. Zadkov

**Affiliations:** 1 Key Laboratory for Physical Electronics and Devices, Ministry of Education, School of Electronic Science and Engineering, Xi’an Jiaotong University, 710049 Xi’an, China; 2 Institute of Spectroscopy, Russian Academy of Sciences, Troitsk, Moscow, 108840 Russia; 3 Quantum Technology Centre, Faculty of Physics, M. V. Lomonosov Moscow State University, Moscow, 119991 Russia; 4 Faculty of Physics, Higher School of Economics, Moscow, 105066 Russia

**Keywords:** Solitons, Nonlinear optics

## Abstract

Higher-order topological insulators (HOTIs) are unique materials hosting topologically protected states, whose dimensionality is at least by 2 lower than that of the bulk. Topological states in such insulators may be strongly confined in their corners which leads to considerable enhancement of nonlinear processes involving such states. However, all nonlinear HOTIs demonstrated so far were built on periodic bulk lattice materials. Here, we demonstrate the first *nonlinear photonic* HOTI with the fractal origin. Despite their fractional effective dimensionality, the HOTIs constructed here on two different types of the Sierpiński gasket waveguide arrays, may support topological corner states for unexpectedly wide range of coupling strengths, even in parameter regions where conventional HOTIs become trivial. We demonstrate thresholdless spatial solitons bifurcating from corner states in nonlinear fractal HOTIs and show that their localization can be efficiently controlled by the input beam power. We observe sharp differences in nonlinear light localization on outer and multiple inner corners and edges representative for these fractal materials. Our findings not only represent a new paradigm for nonlinear topological insulators, but also open new avenues for potential applications of fractal materials to control the light flow.

## Introduction

Fractals are self-similar structures (i.e., next generation of a fractal can be constructed by combining copies of its previous generation) widely represented in universe^1^, whose unusual internal composition finds its manifestation in new physical phenomena observed in solid-state physics, acoustics, and photonics, to mention just a few areas^2–19^. One of the distinguishing characteristics of a fractal is its fractional dimension, which can be described by the non-integer effective Hausdorff dimension ${d}_{f}={\log }_{\ell }m$, where *m* is the number of previous-generation elements required to construct next-generation fractal, while *ℓ* is the factor, by which length of the fractal edge would increase in the next generation. Thus, the famous Sierpiński carpet and gasket fractals have Hausdorff dimensions ${d}_{f}={\log }_{3}8$ and ${d}_{f}={\log }_{2}3$, respectively, which reflects their different composition. Fractal systems are aperiodic, but regular—they are sometimes considered as lacking “bulk” due to the presence of multiple holes, inner edges and corners. In photonics, such unusual composition of fractal structures may open new prospects for manipulation and localization of the light fields in them, including realization of quantum anomalous transport^8^ and flat-band systems^20,21^. Particularly, intriguing problem is the possibility of realization of topological phases in fractal structures, since the appearance of topological edge states is tightly connected with the dimensionality of the system and usually stems from topological properties of its bulk, which can be very specific in fractal systems.

Photonic systems offer a unique testbed for the realization of topologically nontrivial structures^22–24^, including various types of Chern^25^, Floquet^26^, valley-Hall^27^, and higher order^28–33^ topological insulators. Most of these systems, including HOTIs, were constructed on structures with periodic bulk. However, very recently it was shown that unidirectional traveling topological edge states can form in fractal waveguide arrays with helical channels^12,13^ and in fractal Haldane model^34^ implying that formal absence of insulating bulk in fractals is not an obstacle for realization of topological phase, and bulk-edge correspondence^35^ is still meaningful for these systems. While fractal HOTIs were proposed in electronic systems^36,37^ and recently realized in acoustics^14,15^, and in circuits^19^, higher order topological states in *photonic fractals* were never observed so far. At the same time, the first experiments in acoustics hint at a very unusual manifestation of topological effects in fractal systems^14,15^, connected with the possibility of localization in their multiple inner corners, and strong dependence of the parameter range, where fractal system is topological on fractal generation order, making them clearly distinct from conventional HOTIs.

Among the advantages of photonic systems in comparison with the electronic and acoustic ones, is that the former systems can be strongly nonlinear. Nonlinearity not only offers a convenient knob for controlling localization and propagation dynamics of the topological excitations, but it is often crucial for the effects that determine practical applications of such systems^38^, ranging from lasing and harmonic generation to bistability, nonlinearity-controlled switching and routing with the topologically protected states. In addition, nonlinearity gives rise to a broad spectrum of topological edge solitons, inheriting topological protection from their linear counterparts^39–45^ as demonstrated in^46–50^, and it may create the self-induced topological phases^51–53^. Nonlinear effects and formation of the unique corner solitons in HOTIs were recently reported too^54–56^. It is also demonstrated that the nonlinearity may result in the mobility control around the Fermi level^57^ and geometrical frustration^58^.

Nowadays, there is considerable interest in the investigation of nonlinear effects in aperiodic topological photonic systems, with only a few theoretical predictions available so far^59^. Fractal topological photonic systems may provide a unique platform for investigation of such effects, since the behavior of nonlinear modes in fractal lattices with increasing nonlinearity can be surprising and their topological protection is not guaranteed a priori. For example, in topological systems focusing nonlinearity does not necessarily lead to localization enhancement. Nonlinearity allows to tune propagation constants of nonlinear modes within the spectrum of the system, thereby opening the way to tune also the internal structure of excitations depending on their power, which introduces tunability in fabricated topological structures. Thus, topological mode can shift from one gap to another or enter the band under the effect of nonlinearity and this leads to qualitative and complex changes of its internal structure, beyond simple localization or delocalization. The investigation of nonlinear effects in aperiodic topological systems is interesting also from purely fundamental point of view, since this behavior may sharply contrast (due to a much richer spectrum of fractal systems giving rise to richer soliton families and dynamics, see below) with behavior of nonlinear modes in periodic structures.

In this article, we describe the first experimental realization of photonic fractal HOTI and study the interplay between topological and nonlinear effects in this aperiodic system, which gives rise to topological corner solitons. To demonstrate such states we utilize fractal Sierpiński gasket waveguide arrays of two different types inscribed in fused silica using fs laser writing technique^17,26,50,54,56,59–61^. Higher order topological phase is realized due to the controllable shift of the waveguides that adjusts coupling strengths between sites in the first and subsequent generations of fractals and is manifested in the appearance of the corner states of the topological origin. The remarkable distinctive property of this photonic system is that topological states can appear not only in the outer, but also in the inner corners of the structure. Moreover, topological corner states in fractals can exist even in parameter regions, where some of conventional HOTIs with periodic bulk become trivial. To characterize topological properties of this aperiodic system we employ real-space polarization index^62,63^. Finally, we demonstrate thresholdless fractal topological corner solitons bifurcating from their linear counterparts and existing in the forbidden spectral gaps. Notice that in the system considered in this article the Laplacian governing diffraction of light is two-dimensional, while the main unusual properties of the linear spectrum of the system and solitons stem namely from the internal structure of the fractal array. This is in contrast to recently considered lattice systems with fractional Laplacian that affects the very balance between diffraction and nonlinearity that is reflected in properties of solitons in the latter systems^64^.

## Results

### Fractal arrays, their linear spectra and eigenmodes

**Fig. 1:**
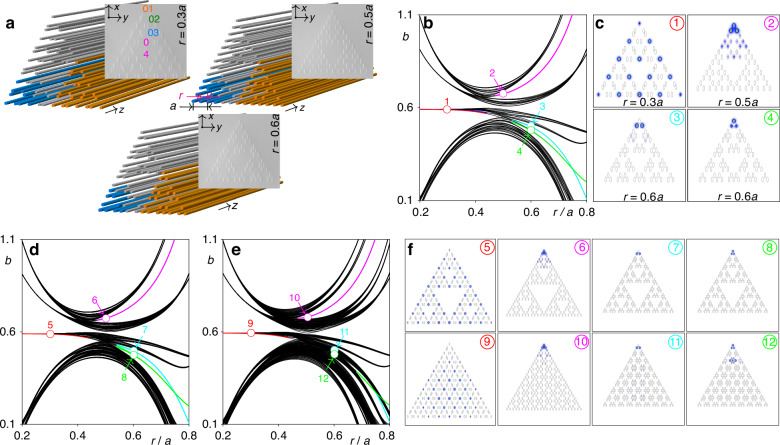
**Fractal higher-order topological insulators and their linear spectra.** **a** Schematic representation of the third generation *G*_3_ of the Sierpiński gasket waveguide arrays with *r* = 0.3*a*, 0.5*a*, and 0.6*a*. Blue and orange sectors of these arrays represent the first-generation *G*_1_ and second-generation *G*_2_ structures, respectively. Microphotographs of the corresponding fs-laser written waveguide arrays are presented in the insets. The orange, green, blue, and magenta ellipses in microphotographs indicate the representative sites 1, 2, 3, and 4 that will be used below for probing of excitation dynamics. **b** Eigenvalues *b* of the stationary linear states of the *G*_3_ fractal array vs distortion parameter *r*. Colored curves represent localized states, while black ones correspond to the delocalized states. **c** Intensity distributions shown within −23 ≤ *x*, *y* ≤ 23 window for four representative eigenstates corresponding to the colored circles in (**b**). **d** Eigenvalues *b* of the stationary linear modes of the *G*_4_ fractal array vs distortion parameter *r*. For comparison, in panel (**e**) we show eigenvalues of linear modes of non-fractal array of the same size as fractal *G*_4_ structure. **f** Intensity distributions shown within −45 ≤ *x*, *y* ≤ 45 window for eigenmodes of *G*_4_ fractal array corresponding to the colored circles 5–6 in (**d**), and for eigenmodes of non-fractal array corresponding to the colored circles 9-12 in (**e**). Here and in figures below the array depth is *p* = 5.7

We consider two different types of Sierpiński gasket arrays in this Article, which are further termed case-1 and case-2 arrays. Both arrays are produced using identical first-generation triangular element *G*_1_ highlighted with the blue color in the schematic representation in Fig. 1a, where we show third generation *G*_3_ of case-2 structure (for more details of fractal array construction and results on case-1 structure see Supporting Information). *G*_*n*_ generation of the Sierpiński gasket fractal array is formed by three *G*_*n*−1_ generation structures (for instance, *G*_2_ elements in Fig. 1a are highlighted with orange color)—on this reason fractal arrays are self-similar. In case-2 arrays these previous generations share three common sites, while in case-1 array there are no such common sites. Consequently, *G*_*n*_ generation includes 3^*n*+1^ sites in case-1 array, and 3^*n*+1^ − (3^*n*^ − 3)/2 sites in case-2 array. Due to the method of their construction, fractal arrays posses multiple holes, inner corners and edges. They are characterized by the effective Hausdorff dimension ${d}_{f}={\log }_{2}3$ that is lower than 2. Further we focus on newly designed case-2 arrays, discussing solitons in the case-1 arrays in Supporting Information. To realize fractal HOTI, we introduce controllable distortion (via the parameter *r*) into the structure by shifting the neighboring waveguides in the opposite directions, while keeping spacing *a* between the next-nearest-neighbor waveguides constant, as indicated in Fig. 1a. The examples of undistorted (*r* = 0.5*a*) and distorted (*r* = 0.3*a* and *r* = 0.6*a*) arrays are shown in Fig. 1a with the photographs of such fs-laser written entities in fused silica.

Propagation of light beams in fractal arrays inscribed in focusing cubic medium can be described by the nonlinear Schrödinger equation:

1 $$i\frac{\partial \psi }{\partial z}=-\frac{1}{2}\left(\frac{{\partial }^{2}}{\partial {x}^{2}}+\frac{{\partial }^{2}}{\partial {y}^{2}}\right)\psi -{\mathcal{R}}(x,y)\psi -| \psi {| }^{2}\psi$$

where *ψ* is the dimensionless complex amplitude of the light field; *x*, *y,* and *z* are the normalized transverse coordinates and propagation distance, respectively; the function ${\mathcal{R}}(x,y)=p{\sum }_{mn}{e}^{-{(x-{x}_{m})}^{2}/{d}_{x}^{2}-{(y-{y}_{n})}^{2}/{d}_{y}^{2}}$ describes the fractal case-2 waveguide array composed of single-mode elliptical (due to writing process) waveguides with the depth *p* and widths *d*_*x*,*y*_ placed in the nodes (*x*_*m*_, *y*_*n*_) of the Sierpiński gasket grid. Further, we use arrays with *p* = 5.7, *a* = 6.0, and *d*_*x*_ = 0.25, *d*_*y*_ = 0.75 corresponding to the parameters of the experimental structures (see Materials and Methods for details of normalization). Among the advantages of continuous model Eq. (1) is that it takes into account the exact shapes of the waveguides (sites of the array), accounts for coupling between all waveguides of the structure, even very distant ones, possible radiation from waveguides, and it even describes the variation of modal shapes inside the waveguides that can be caused by the nonlinearity and that may affect stability (for example, in tight-binding model operating with modal amplitudes, the dynamics of field inside waveguides is not considered). At the same time, tight-binding Hamiltonian that can be derived from continuous model (1) sometimes allows analytical treatment of the system and is particularly helpful for the characterization of its topological properties. Therefore, we further mainly use the continuous model to reproduce experimental results and to accurately describe the main distinctive features of linear spectrum of the system and use its tight-binding version as an auxiliary tool for the characterization of topological properties. Notice that model (1) possesses time-reversal symmetry, while if one disregards the ellipticity of the waveguides, the corresponding array is characterized also by ${{\mathcal{C}}}_{3}$ discrete rotational symmetry.

We first characterize the linear spectrum of such arrays by omitting nonlinearity in Eq. (1) and calculating the linear eigenmodes of the form *ψ* = *u*(*x*, *y*)*e*^*ibz*^ using plane-wave expansion method (see Materials and Methods), where *u* describes the modal shape, and *b* is the propagation constant (eigenvalue). The linear spectrum of the fractal array of third generation *G*_3_ in the form of dependence of eigenvalues of all supported modes *b* on distortion parameter *r* is presented in Fig. 1b, where colored curves correspond to the localized states, while black curves correspond to the extended states. Remarkably, in fractal arrays localized in-gap states are encountered in both *r* > 0.5*a* and *r* < 0.5*a* regimes, in sharp contrast with some HOTIs with periodic bulk (like kagome or square SSH lattices), where localized states appear only for one of these types of distortion leading to dominance of the inter-cell coupling over the intra-cell one. Notice that the state shown with magenta curve can exist even at *r* = 0.5*a*. Intensity distributions of four typical localized states corresponding to circles in Fig. 1b are shown in Fig. 1c. Magenta, green, and cyan branches correspond to the co-existing *outer corner states* with different internal structure, representative namely for the case-2 fractal array (notice that corner modes should not necessarily have light in the very corner waveguide, see for example mode 3 that has two out-of-phase spots in nearest to corner waveguides and that coexists with mode 4, whose main maximum is located in the corner). Co-existence of several corner modes is also known in non-fractal systems^29^. In contrast, red branch corresponds to the states, where strongly localized spots appear simultaneously in multiple inner and outer corners, so one can call them *hybrid corner states*, as they reflect unique internal composition of the fractal array (there are several such branches in spectrum with spots only in the corners that become nearly degenerate for small *r* values, see mode 1 in Fig. 1c). Outer corner states are all three-fold degenerate, while their number is not affected by the fractal generation order *n*. According to the definition of the effective dimensionality ${d}_{e}={\lim }_{n\to \infty }(\ln N/\ln {N}_{l})$, where *N* is the number of sites occupied by the corner state and *N*_*l*_ = 3 ⋅ 2^*n*−1^ + 1 is the total number of sites on one outer edge^14,15^, outer corner states are zero-dimensional, as *d*_*e*_ → 0 with increase of the fractal generation order *n*. In contrast, the number of spots in hybrid corner states *N* = (3^*n*^ + 3)/2 increases with *n*, so that *d*_*e*_ for hybrid state approaches effective Hausdorff dimensionality ${d}_{f}={\log }_{2}3$ of the structure. Thus, fractal HOTIs offer, in principle, the opportunity to observe linear photonic corner states of different effective dimensionality. However, while excitation of outer corner states with *d*_*e*_ → 0 is technically simple because it can be achieved by focusing light into several corner waveguides only, the observation of truly stationary linear hybrid corner states with *d*_*e*_ → *d*_*f*_ may be more challenging and would require simultaneous excitation of many sites, including inner corners, as in mode 1 from Fig. 1c, to avoid slow switching of light into non-excited sites. Notice that such switching can be suppressed even by very weak nonlinearity that allows us to observe even strongly localized hybrid corner solitons.

To illustrate that the spectrum of the system remains qualitatively similar for fractals of different generations and to prove that corner states in fractal topological insulators are indeed well-localized, in Fig. 1d we present a linear spectrum of the fourth generation *G*_4_ of the Sierpiński gasket waveguide array. Remarkably, while the density of lines in bands of delocalized states has increased in Fig. 1d in comparison with spectrum Fig. 1b of *G*_3_ system, the structure of the spectrum did not change, and all corner states associated with magenta, cyan, green lines, and hybrid corner states associated with red line are clearly visible in the spectrum. The examples of such corner states are presented in the top row of Fig. 1f. One can clearly see that states 6-8 are localized in the outer corner of the structure, while hybrid state 5 has intensity maxima also in all inner corners. This confirms that the formation of corner states in fractal structure is a robust effect that persists in all generations. For the sake of comparison, in Fig. 1e we present also linear spectrum of *non-fractal* waveguide array of the same size as *G*_4_ structure. One can observe that multiple additional bands appear in the spectrum of this system (particularly at *r* > 0.5*a*) in comparison with the fractal structure. While states associated with red and magenta curves remain practically unaffected, green and cyan corner state branches strongly overlap with newly emerged bands, i.e. the existence domains of associated corner states in *r* are strongly reduced. Indeed, profiles of corresponding modes in points 11 and 12 at *r* = 0.6*a* presented in the bottom row of Fig. 1f reveal coupling with bulk modes, while in fractal system similar states 7 and 8 are strongly localized in the corner. This hints on the fact that fractality of the structure not only preserves nontrivial topology (as was concluded for Chern insulators supporting unidirectional edge states^12,13^), but even substantially expands the domain of existence of certain topological states. It should also be mentioned that even though the counterpart of hybrid red branch in the fractal array exists in non-fractal system (due to the very structure of *G*_1_ element), the effective dimensionality of the latter state is 2 as opposed to the effective Hausdorff dimensionality ${d}_{f}={\log }_{2}3$ of this state in fractal system.

We also compared the results of dynamical single-site excitation (in linear case) of representative sites in fractal and non-fractal *G*_4_ structures (see Supporting Information). It was found at *r* < 0.5*a* the output patterns for corner excitations are similar as in both cases strongly localized corner states are excited. The largest differences between diffraction patterns in fractal and non-fractal geometries are observed at *r* = 0.5*a*, where diffraction in the non-fractal system is usually stronger. Finally, at *r* > 0.5*a* the excitation of outer corners in both geometries leads to the formation of topological corner modes, but dynamics in the bulk can be different. These results illustrate that the fractality of the structure does not destroy the HOTI phase.

To characterize the topological properties of fractal Sierpiński gasket waveguide arrays one can employ the real-space polarization index^62,63^. This is because fractal structure considered here is aperiodic and lacks translational symmetry^65^. The nonzero quantized polarization index together with the rotational crystalline symmetry is used to predict the appearance of the corner states^32^. The remarkable prediction of this analysis (to calculate real-space polarization index we glue two Sierpiński gaskets to form a rhombic structure, and then set to zero couplings with “missing” sites to reproduce fractal system—see Materials and Methods and Supporting Information for details) is that this index for both hybrid and outer corner states is 0.5 in their respective existence domains within the gap, meaning that all such corner states are of topological origin. Similar conclusions can be drawn for the case-1 fractal arrays, as we also discuss in Supporting Information. Thus, fractal Sierpiński gasket arrays allow to realize HOTIs in a broader range of distortion parameters *r* in comparison with HOTIs based on the periodic kagome and Su-Schrieffer-Heeger arrays^54,55^, where in our notations corner states are possible only at *r* < 0.5*a*. Note that although the topological nature of corner modes in tight-binding triangular and kagome-like structures has been discussed^66,67^, recent theoretical and experimental studies in various fields of physics^32,54,68–73^ show that in the presence of disorder such structures provide absolutely the same degree of topological protection as systems based on square or honeycomb lattices. In the presence of a small disorder in the depths and positions of the waveguides, the eigenvalues of the corner modes may fluctuate slightly, but they remain in the topological gap and the topological protection (localization and absence of coupling with bulk modes) is preserved as long as the introduced disorder is not strong enough to close/completely destroy the topological gap. The topological modes in our fractal system also show this resilience to disorder. Taking into account that our system features time-reversal symmetry, while the array profile possesses ${{\mathcal{C}}}_{3}$ discrete rotational symmetry (if one disregards non-crucial ellipticity of the waveguides), one can conclude that our system can be classified as a higher order topological crystalline insulator^32,74–78^, which shows the absence of quantized multipole moments but is topologically protected by the ${{\mathcal{C}}}_{3}$ rotational symmetry.

Thus, summarizing the above properties of the spectrum of fractal structures in comparison with other types of lattices already used for the construction of HOTIs (including SSH and kagome ones) one can conclude that (i) the fractal arrays can be topological in both regimes *r* < 0.5*a* and *r* > 0.5*a*, and that topological states in them can appear with much richer shapes allowing to create topological corner solitons with multipole internal structure (see modes 6–8 in Fig. 1); (ii) several topological states can co-exist in spectrum of fractal arrays for the same value of parameter *r* and give rise to different stable soliton families; (iii) hybrid topological states can appear in any internal corner of the structure that can be important for potential practical applications (see modes 5 and 9 in Fig. 1); and finally, lack of insulating bulk in fractal system may even broaden the range of existence of some topological states existing in non-fractal geometry.

### Topological soliton families

In the presence of nonlinearity localized corner states in fractal arrays give rise to the families of bifurcating from them thresholdless topological corner solitons. Because in our experimental system that we model here temporal dynamics can be neglected due to the long pulses used, we consider only the competition between diffraction, refraction in inhomogeneous landscape ${\mathcal{R}}$, and focusing nonlinearity. Thus, solitons that we consider here are spatial solitons. They can emerge in the gaps of the linear spectrum from different topological corner states and they actually represent nonlinear deformations of these states, but this deformation may be strong, because sufficiently strong nonlinearity can lead to contraction, or instead to strong expansion of the corner soliton. Such spatial solitons also have the form *ψ* = *u*(*x*, *y*)*e*^*ibz*^, whose substitution into Eq. (1) yields the equation $bu=\frac{1}{2}({\partial }_{x}^{2}+{\partial }_{y}^{2})u+{\mathcal{R}}u+{u}^{3}$ that was solved using Newton method (see Materials and Methods). Now propagation constant *b* parameterizes the family of spatial solitons, determining their power *U* = ∫∫∣*ψ*∣^2^*dxdy*. In the bifurcation point from linear topological corner state the propagation constant of spatial soliton *b* coincides with the eigenvalue of corresponding linear state *b*_lin_. The amplitude $\max | u|$ and power *U* of soliton vanish as *b* → *b*_lin_, i.e. in the bifurcation point the soliton transforms into a corresponding linear state with vanishing amplitude. On this reason, the solitons naturally inherit the internal structure of linear corner states from which they emerge. This also means that such solitons are thresholdless, i.e. they exist even at low powers. When *b* increases away from the bifurcation point, the amplitude and power of soliton increase indicating on progressively growing impact of nonlinearity that of course considerably affects the shapes of spatial solitons.

**Fig. 2:**
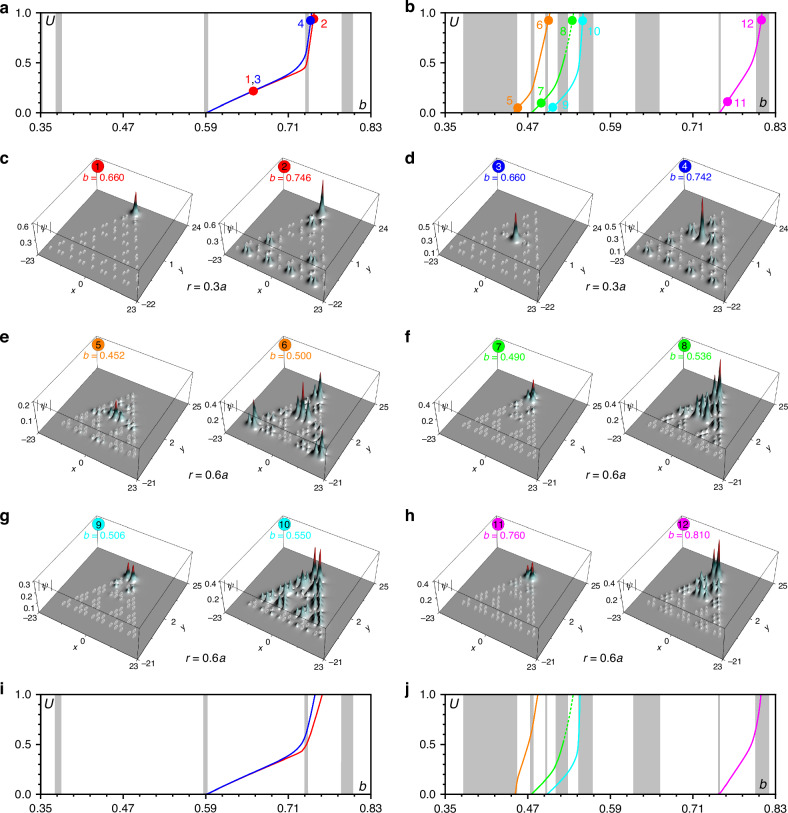
**Topological solitons in fractal insulators**. **a** Power vs propagation constant for the solitons bifurcating from the linear eigenstates concentrated around sites 1 (red curve) and 4 (blue curve) in the fractal array with *r* = 0.3*a*. Panels (**c**) and (**d**) show field modulus distributions corresponding to the dots in (**a**). **b** Power vs propagation constant for the solitons bifurcating from linear eigenstates concentrated around sites 1 (green, cyan and magenta curves) and 4 (orange curve) in fractal array with *r* = 0.6*a*. Field modulus distributions corresponding to the dots in (**b**) are shown in (**e**)–(**h**). In (**a**) and (**b**) gray regions are associated either with the eigenvalues of the localized states or with the bands of the delocalized states. **i**, **j** Power vs propagation constant for solitons in *G*_4_ fractal structure that correspond to the same types and locations of solutions as in panels (**a**),(**b**) that are for *G*_3_ fractal structure. Representative profiles of solitons in *G*_4_ structure are shown in Supporting Information

The families of corner solitons in fractal insulators can be very rich. In Fig. 2 we show representative *U*(*b*) dependencies in *G*_3_ structures. At *r* = 0.3*a*, when a set of practically degenerate linear topological modes with spots localized only on the inner and outer corners exist, topological solitons can form in any corner of the fractal array, see red (outer corner) and blue (inner corner) families in Fig. 2a bifurcating from corresponding linear modes (or their linear combination, because for this *r* there exist a set of practically degenerate *hybrid corner states*). Propagation constants *b*_lin_ ≈ 0.59 of such linear hybrid corner states from which soliton bifurcation occurs are indicated in Fig. 2a by a narrow gray region. Notice that red and blue *U*(*b*) soliton branches may be very close, especially at *b* → *b*_lin_, but they correspond to different soliton solutions located in outer and inner corners of the array, respectively, as seen from profiles in Fig. 2c, d. For selected *r* value such spatial solitons are strongly localized at the low and intermediate powers (see profiles in Fig. 2c, d corresponding to points 1 and 3 in Fig. 2a). For illustrative purposes here we superimpose ∣*ψ*∣ distribution for spatial soliton on array profile ${\mathcal{R}}$ shown by white ellipses. Importantly, because nonlinearity leads to shift of the propagation constant within the gap of the linear spectrum, one can control the localization degree of corner solitons by increasing their power. The transformation of spatial soliton shape substantially depends on where the propagation constant of the linear corner state giving rise to the soliton family is located in the gap. If it is located close to the bottom of the gap, the spatial soliton may first contract and then expand, when its propagation constant approaches the band of extended states. If the linear corner state is located close to the center of the gap and is already sufficiently well localized, the soliton tends to broaden under the action of focusing nonlinearity when its propagation constant approaches the band. When *b* enters into the band occupied by extended states (and even crosses it), the spatial soliton couples with bulk modes and looses localization (as illustrated by profiles in points 2 and 4 of Fig. 2a, where soliton clearly couples with bulk states). It should be stressed though, that such spatial solitons coupled to bulk modes are still self-sustained nonlinear modes, and even though they extend across the array, they can still be dynamically stable. It should be stressed that in focusing nonlinear medium considered here hybridization of solitons with extended states can occur only for linear bands of extended states (shown in Fig. 2a, b with wider gray domains) laying to *the right* of the propagation constant *b*_lin_ of linear corner state, from which soliton bifurcates. This is because focusing nonlinearity tends to increase *b*. Bands of extended states laying to the left of *b*_lin_ are therefore not excited, as nonlinearity shifts the propagation constant of soliton further away from these bands.

Fractal HOTI with *r* = 0.6*a* supports multiple families of topological solitons with different symmetries forming in the *outer corners*. They are shown in Fig. 2b with magenta, cyan, and green lines. When the power *U* for these families of solitons shown by different colored lines vanishes, their propagation constants approach different vertical gray lines or narrow regions corresponding to propagation constants *b*_lin_ of linear corner states. These propagation constants are also indicated in Fig. 1b by lines of the same color as for soliton families. Solitons inherit the symmetry of the corresponding linear states. Thus, in the green family (Fig. 2f, points 7 and 8) corner maximum is out-of-phase with maxima in two nearest-neighbor sites; solitons from blue family have empty outer corner site, while fields in nearest-neighbor sites are out-of-phase (Fig. 2g, points 9 and 10); while most localized solitons from magenta branch have nearly equal intensities in three close sites in the corner (Fig. 2h, points 11 and 12). While the internal structure of soliton solutions is determined by the structure of linear modes giving rise to corresponding nonlinear families (making them looking as dipoles as in Fig. 2g or combinations of in-phase humps as in Fig. 2h), such states cannot be represented as nonlinear combinations of several simpler (single-hump) solitons, because there are no such more localized and thresholdless states in corresponding topological gaps that would occupy only one of involved waveguides. For selected *r* value such solitons typically gradually broaden with increase of power and strongly expand into array when coupling with extended states occurs upon entering their propagation constant *b* into different bands of the linear spectrum. This coupling dramatically changes the structure of nonlinear modes. Interestingly, for most of these solutions one can find in the next gap (lying above the gap where bifurcation from linear corner mode occurs) a family that looks like a “continuation” of the family in lower gap. Corresponding solutions usually have different symmetries of tails. Notice also that extended linear states occupying multiple sites can also produce nonlinear families, as illustrated by the orange branch in Fig. 2b, see corresponding nonlinear modes in Fig. 2e. As shown in Fig. 2b, this branch bifurcates from the top of the band of extended states. While this branch does not cross with branches of corner solitons (green or cyan curves), with increase of its power one can see certain growth of intensity in the corners of the structure in such extended states (point 6, Fig. 2e).

Notice that nonlinearity, whose impact increases with increase of soliton power *U*, affects the symmetry of the total refractive index landscape ${\mathcal{R}}(x,y)+| \psi {| }^{2}$ in a way determined by the shape of particular nonlinear topological state *ψ*. When the nonlinear state is in the gap, it is still topologically protected, but when nonlinearity becomes strong enough to shift its propagation constant into the band, one may conclude that the perturbation induced by nonlinearity became sufficiently strong to destroy the protection. It is thus reasonable to consider the power at which nonlinear family enters into the band of extended states as a critical power for this particular type of solution at which it looses protection.

It should be stressed that the properties of topological solitons do not change qualitatively in larger fractal systems, i.e. our results remain valid for any fractal generation (this is also the consequence of the fact, that the linear spectrum of the fractal array remains qualitatively similar in next generation, see Fig. 1d). To illustrate this, we obtained all soliton families presented in Fig. 2a, b for *G*_3_ structure, also in larger *G*_4_ array, see Fig. 2i, j and Supporting Information. One can see that the behavior of soliton families *U*(*b*) in larger structure is qualitatively similar: Increasing power results in reshaping of the nonlinear states and eventually drives them into the band (different families feature different degree of localization, but inside the gap they all are localized near respective corners). Stability properties also remain similar in *G*_4_ array, see solid branches in Fig. 2i, j corresponding to stable solitons, and the only dashed green branch corresponding to unstable states.

We also note that families of topological corner solitons can exist in non-fractal HOTIs. This becomes clear from a comparison of linear spectra of fractal and non-fractal arrays in Fig. 1d, e. Thus, soliton families bifurcating from red and magenta curves in Fig. 1e will share similar properties for all values of parameter *r* with fractal soliton families (of course, there will be some differences in soliton shapes dictated by different internal structures of corresponding arrays). At the same time, some other families of localized corner states will actually disappear because corresponding linear modes in non-fractal geometry couple with bulk modes (as it happens for green and cyan curves in Fig. 1e that at least for *r* = 0.6*a* overlap with bulk band). We found that solitons bifurcating from a red branch at *r* = 0.3*a* and from a magenta branch at *r* = 0.6*a* are stable in non-fractal geometry.

Despite a large variety of corner solitons appearing in fractal HOTI, nearly all of them are dynamically stable. Their stability was verified by adding small-scale random perturbation (up to 5% in the amplitude) into input field distributions and propagating them in the frames of Eq. (1) over a very long distance *z* ~ 10^4^ exceeding length of our sample *z* = 88 (that corresponds to 10 cm) by two orders of magnitude. This is sufficient for the detection of all possible, even very weak instabilities. For all branches shown in Fig. 2a, b with solid lines, such perturbations resulted only in small-amplitude oscillations signalizing on their stability, while decay was observed only for a small part of the green branch shown with the dashed line. To provide even more rigorous proof of existence of stable corner solitons in fractal HOTI, we performed linear stability analysis for all obtained soliton branches. The results of the stability analysis described in Supporting Information are fully consistent with results obtained by direct propagation of perturbed states.

### Observation of the corner solitons in fractal HOTI

To demonstrate corner solitons in fractal HOTIs we fabricated a set of case-2 *G*_3_ structures with various distortion parameters *r* = 0.3*a*, *r* = 0.5*a*, and *r* = 0.6*a* using fs-laser inscription technique (for experiments with case-1 array see Supporting Information). Arrays were inscribed in 10 cm-long fused silica samples (see Materials and Methods for details of fabrication). Exemplary photographs of the inscribed arrays are presented as insets in Fig. 1a. We selected four representative locations to study excitation dynamics, as indicated with colored ellipses with numbers 1 (outer corner site), 2 (site on the outer edge), 3 (site on the inner edge site) and 4 (inner corner site). For excitation we used pulses with a duration of about 280 fs of variable energy *E* derived from 1 kHz Ti:sapphire laser to achieve strong nonlinear response (see Materials and Methods).

**Fig. 3:**
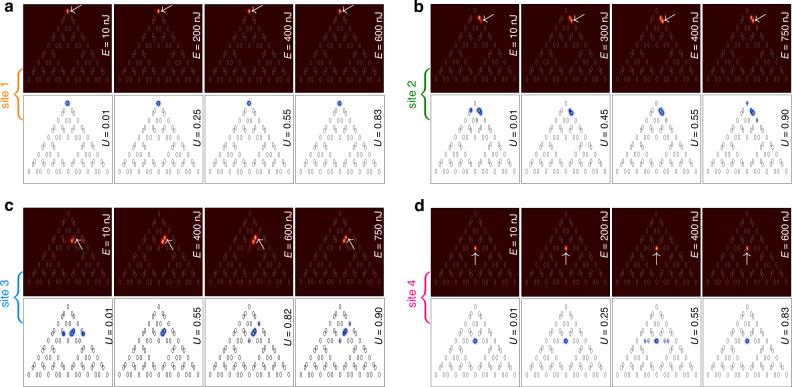
**Observation of nonlinear states in fractal array with** *r* = 0.3*a*. Output intensity distributions after 10 cm of propagation for the excitation of sites 1 (**a**), 2 (**b**), 3 (**c**) and 4 (**d**) in *G*_3_ fractal array. Figures with the maroon background show experimental results, while figures with the white background show results of theoretical simulations. Gray ellipses in all panels indicate waveguide locations. Input pulse energies *E* and powers *U* are indicated on each plot. All distributions are shown within the window − 23≤*x*, *y*≤23. Arrows in the experimental panels indicate position of the input excitation

First, we investigate the structure with *r* = 0.3*a* that supports *hybrid corner states*. In Fig. 3a–d, we compare experimental output intensity distributions (images with maroon background) obtained at the different pulse energies *E* for the excitation of the four above-mentioned sites of the array (in each case, the excited sites are indicated by white arrows) with theoretical distributions for the different powers *U* obtained by solving Eq. (1) (images with white background) Here, we utilize the split-step Fourier method for solving Eq. (1) with a given input (see Materials and Methods). Notice that because pulsed excitations are used, output experimental intensity distributions represent averaged patterns containing contributions from regions around pulse peak, propagating in the strongly nonlinear regime, and contributions from linearly diffracting tails, that usually make the averaged pattern less localized, slightly washing out transitions from delocalized to localized patterns upon variation of pulse energy *E*. Also, the output may slightly vary depending on the efficiency of coupling of focused light beam into the selected waveguide. Thus, when experiments are compared with the results of modeling, the power level *U* in simulations is selected for each case to produce the best agreement with the experimental distribution. Since well-localized thresholdless solitons at *r* = 0.3*a* can form in any inner or outer corner of this structure, the excitation of sites 1 and 4 (Fig. 3a, d) yields strongly localized, practically single-site patterns in both linear (*E* = 10 nJ) and nonlinear (e.g., *E* = 750 nJ) regimes, confirming the formation of thresholdless corner solitons. In contrast, when exciting edge sites 2 and 3 (Fig. 3b, c), we observe diffraction and dynamic oscillations of power between close pairs of waveguides even at pulse energies *E* ~ 800 nJ, indicating that inner and outer edges do not support well-localized thresholdless in-gap states and significant power levels are needed to achieve localization at such edges. It should also be stressed that while theory predicts soliton expansion due to coupling with bulk modes at sufficiently large power levels, it is hard to achieve corresponding pulse energies at which this coupling is visible in the experiment without producing optical damage to the material, especially at the input facet. In this system, where the width of the topological gap is not small (especially at *r* = 0.3*a*), crossing of the gap requires pulse energies about *E* ~ 1000 nJ and this is already very close to the optical damage threshold. We thus present the results for pulse energies, at which material and waveguiding structure cannot be damaged.

**Fig. 4:**
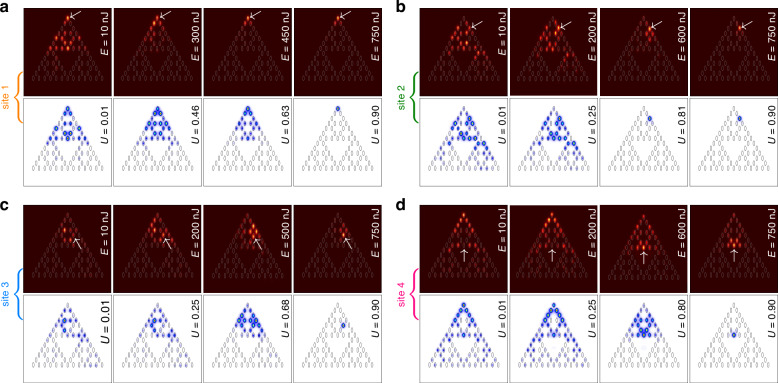
**Nonlinear dynamics in fractal array with** *r* = 0.5*a*. Rich nonlinear dynamics and gradual transition to the localization at the highest power levels as observed for the excitations of sites 1-4 in *G*_3_ fractal array. The arrangement of panels is the same as in Fig. 3. Arrows in experimental panels indicate position of the input excitation

Turning to an array with *r* = 0.5*a*, where spacing between nearest sites is the same in the entire array, one should take into account that even though the magenta branch of outer corner states already exists in this borderline case, its localization is rather weak (see state 2 in Fig. 1c), thus the efficiency of its excitation with single-site input is low. On this reason, in Fig. 4a showing experimental patterns for this structure, the beam with *E* = 10 nJ launched into site 1 experiences diffraction, even though some fraction of power clearly remains in the corner waveguide. Since the width of the gap for *r* = 0.5*a* case is rather narrow, even moderate variations of pulse energy may cause considerable variations of the width of the output intensity distribution. Thus, diffraction becomes nearly suppressed when the pulse energy increases to the moderate value of *E* = 450 nJ, while further increase of *E* results in the excitation of the outer corner soliton. For excitation of sites 2, 3, and 4 shown in Fig. 4b–d, respectively, one needs substantially higher pulse energies to achieve comparable degree of localization, while particularly for sites 3 and 4 localization remains weak even for the energies of *E* ~ 750 nJ.

**Fig. 5:**
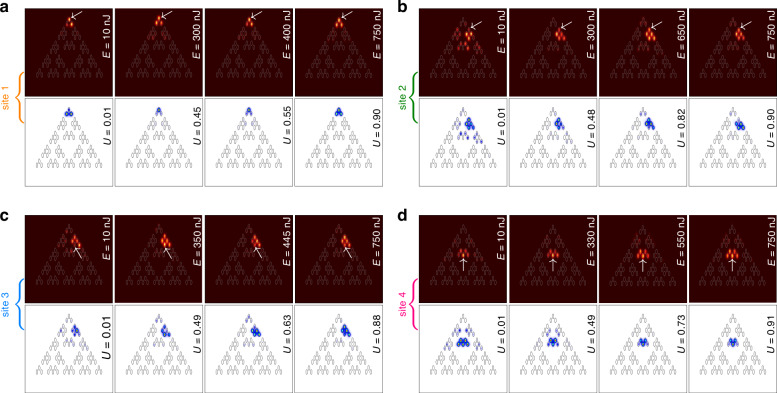
**Observation of nonlinear states in fractal array with** *r* = 0.6*a*. Formation of the localized thresholdless nonlinear states for the excitation of sites 1 (**a**) and 4 (**d**) and rich nonlinear dynamics with the light switching between several closely located waveguides for excitation of sites 2 (**b**) and 3 (**c**) observed in *G*_3_ fractal array. Arrows in the experimental panels indicate position of the input excitation

Fractal case-2 array with *r* = 0.6*a* supports three types of outer corner solitons with different symmetries, as shown in Fig. 2b. Among them, the soliton belonging to cyan branch cannot be excited by focusing beam into site 1, because this site is empty for such nonlinear states (due to their parity). Therefore, excitation of site 1 is supposed to yield nonlinear combination of states from magenta and green branches of Fig. 2b. This is what we observe in Fig. 5a, where for all pulse energies from 10 to 750 nJ light remains practically confined in three closely spaced outer corner waveguides, closely resembling the profile of the outer corner soliton and confirming that in this case it is also thresholdless. Notice that in this case, as in the *r* = 0.3*a* case, it was also impossible to reach pulse energies at which coupling with bulk modes becomes pronounced without optical damage of the material. For *r* = 0.6*a* no localized linear states exist in the array, except for states in outer corners. Consequently, excitations of sites 2 and 3 at all pulse energies yield broad output distributions, where power oscillates between five waveguides, and where intensity maximum may not be located in the excited waveguide (Fig. 5b, c). Similarly, excitation of site 4 yields nonlinear state akin to nontopological state from orange branch of Fig. 2b. Interestingly, with increase of *E* this state initially slightly contracts, but then expands as seen in Fig. 5d (compare this state with well-localized inner corner soliton obtained in the same location at *r* = 0.3*a*).

**Fig. 6:**
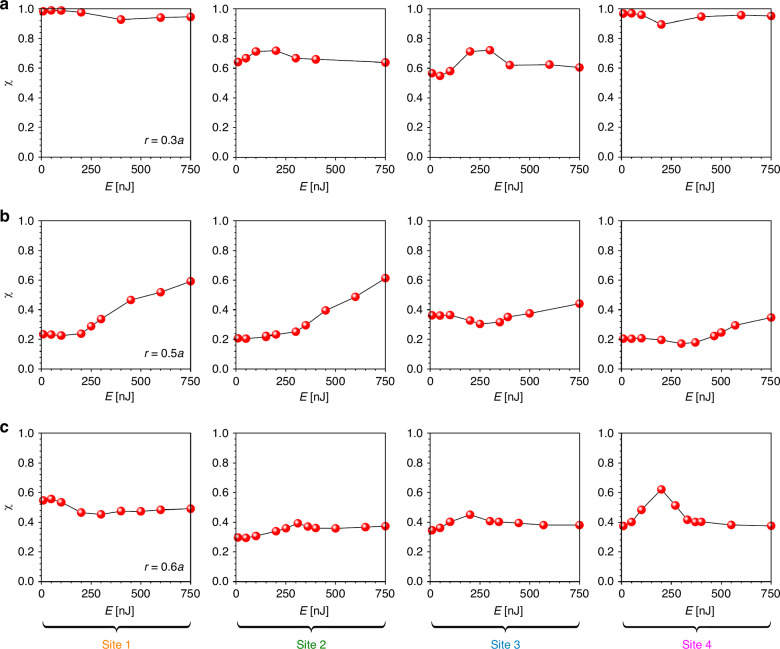
**Experimentally measured output form-factor**. The form-factor as a function of pulse energy in case-2 arrays with *r* = 0.3*a* (**a**), *r* = 0.5*a* (**b**), and *r* = 0.6*a* (**c**). First to fourth columns correspond to excitations of sites 1 to 4, respectively. Standard deviation of measured form-factor does not exceed the size of red dots

To quantitatively characterize the localization of all output intensity distributions presented in Figs. 3–5 in Fig. 6 we show the dependence of the experimentally measured form-factor *χ* as a function of input pulse energy *E* for all considered values of *r* and excitation positions. Here the form factor is calculated as $\chi ={[\iint\,{I}^{2}{\rm{d}}x{\rm{d}}y/{(\iint\,I{\rm{d}}x{\rm{d}}y)}^{2}]}^{1/2}$, where *I*(*x*,*y*) is the measured output intensity distribution. Large *χ* ~ 1 implies good localization, while low *χ* values correspond to delocalized outputs, since this quantity is approximately inversely proportional to the width of the pattern. In Fig. 6a at *r* = 0.3*a* one clearly sees that for excitation of sites 1 and 4 hosting hybrid corner modes *χ* remains close to 1 for all energy levels indicating on very strong localization of the beam. When sites 2 and 3 are excited, no topological modes form and form-factor is notably reduced, but because at this distance the light beam oscillates mainly between two waveguides, *χ* drops down only to ~ 0.6. In Fig. 6b at *r* = 0.5*a* the excitation efficiency of corner state at site 1 is very low, hence *χ* is small (~ 0.2) in linear regime, but it substantially increases with *E* reflecting the fact of nonlinearity-induced contraction of state to corner waveguide observed in Fig. 4a. Similar nonlinearity-induced localization is observed for excitation at site 2, but for all other excitation positions we do not observe pronounced localization at available energy levels. Finally, in Fig. 6c at *r* = 0.6*a* excitation at site 1 yields combination of two topological corner states that is reflected in moderate form-factor *χ* ~ 0.55 because light remains concentrated approximately on three waveguides at all energy levels. For excitation of sites 2-4, when light oscillates between five closely spaced waveguides and no topological states form, one again observes relatively low *χ* values for all power levels, with the only exception for site 4, where around *E* ~ 200 nJ nonlinearity does cause certain contraction of the output, visible also in Fig. 5d in both experiments and simulations. These results are in full agreement with theoretical simulations of dynamical excitation of selected sites.

## Discussions

In summary, we have reported the first example of nonlinear photonic fractal HOTI that supports a rich variety of topological corner states. The remarkable new feature of fractal structures considered here is that they possess corner states (that may have different effective dimensionality) for a very broad range of distortion parameters, substantially exceeding the range, where higher order topological phase emerges in HOTIs built on periodic lattices. The presence of such states facilitates nonlinear light localization and resulted in observation of thresholdless corner solitons, in both outer and inner corners of these structures. Our results extend the class of HOTIs and highlight new prospects for exploration and practical utilization of nonlinear phenomena in photonic fractals. They may be used in new designs of topological lasers or on-chip lasers^79^ that can potentially emit in richer set of states than conventional higher order topological lasers (for example, depending on gain landscape and amplitude, lasing can occur either in outer or in inner corners of the structure, or in corner states with different parity), in shaping of higher harmonic fields in various parametric processes, design of fractal microresonator networks and quantum interfaces of fractal structures, control of condensation in light-matter systems with strong coupling, like polariton condensates in fractal microcavities under resonant or nonresonant pump, and in many other settings. Last but not least, the results are not limited to the optical waveguide array systems; they may inspire related investigations in metasurfaces^80^ or thermal photonic systems^81^.

## Materials and methods

### Normalization of parameters in theoretical model

The transverse coordinates *x*, *y* in Eq. (1) are normalized to the characteristic scale *r*_0_ = 10 *μ*m, the propagation distance *z* is normalized to the diffraction length $k{r}_{0}^{2}\approx 1.14\,{\rm{mm}}$ (corresponding to *z* = 1), where *k* = 2*πn*/*λ* is the wavenumber in the medium with unperturbed refractive index *n* (for fused silica *n* ≈ 1.45 and the nonlinear refractive index *n*_2_ ≈ 2.7 × 10^−20^ m^2^/W), and *λ* = 800 nm is the working wavelength. The array depth $p={k}^{2}{r}_{0}^{2}\delta n/n$ is proportional to the refractive index contrast *δn* of the waveguides. Thus, in our arrays next-nearest-waveguide distance *a* = 6.0 corresponds to 60 *μ*m, waveguide widths *d*_*x*_ = 0.25, *d*_*y*_ = 0.75 correspond to 2.5 *μ*m × 7.5 *μ*m wide elliptical waveguides, sample length of 10 cm corresponds to *z* ≈ 88, while array depth *p* = 5.7 corresponds to refractive index contrast *δn* ≈ 6.4 × 10^−4^.

### The plane-wave expansion method for calculation of linear spectrum

By inserting the ansatz *ψ* = *ue*^*ibz*^ into Eq. (1), one obtains the equation

2 $$bu=\frac{1}{2}\left(\frac{{\partial }^{2}}{\partial {x}^{2}}+\frac{{\partial }^{2}}{\partial {y}^{2}}\right)u+{\mathcal{R}}u+{u}^{3}$$

Here, we choose the fractal array ${\mathcal{R}}$ shown in Fig. 1a as a supercell for the plane-wave expansion method. We expand *u* and ${\mathcal{R}}$ into the Fourier series with the sufficient number of harmonics:

3 $$u=\sum _{m,n}{c}_{m,n}{e}^{i{K}_{m}x+i{K}_{n}y},\,{\mathcal{R}}=\sum _{l,s}{v}_{l,s}{e}^{i{K}_{l}x+i{K}_{s}y}$$

where *c*_*m*,*n*_ and *v*_*l*,*s*_ are the Fourier coefficients, *K*_*m*,*l*_ = 2(*m*, *l*)*π*/*D*_*x*_, *K*_*n*,*s*_ = 2(*n*, *s*)*π*/*D*_*y*_, *D*_*x*,*y*_ are the sizes of the supercell along the *x*, *y* axes, and (*m*, *n*, *l*, *s*) are integers. Plugging Eq. (3) into the linear version of Eq. (2), after simple algebraic transformations one obtains a series of linear equations with different (*m*, *n*, *l*, *s*):

4 $$-\frac{1}{2}\left({K}_{m}^{2}+{K}_{n}^{2}\right){c}_{m,n}+\sum _{l,s}{v}_{l,s}{c}_{m-l,n-s}=b{c}_{m,n}$$

Rewriting Eq. (4) in matrix format and diagonalizing the matrix, one obtains the eigenvalues *b* (i.e. the spectrum) and the corresponding eigenvectors *c*_*m*,*n*_ that allow to construct the eigenmodes *u* of the array according to Eq. (3).

### The Newton method for calculation of nonlinear states

To obtain topological corner solitons we transform Eq. (2) with included nonlinear term into a series of nonlinear equations *f*_*m*,*n*_ = 0 using the finite-difference approximation of derivatives:

5 $$\begin{array}{rcl}{f}_{m,n}\,({\bf{u}})\,=\,\frac{1}{2}\left(\begin{array}{l}\frac{{u}_{m+1,n}\,-\,2{u}_{m,n}\,+\,{u}_{m-1,n}}{d{x}^{2}}+\\ \frac{{u}_{m,n+1}\,-\,2{u}_{m,n}\,+\,{u}_{m,n-1}}{d{y}^{2}}\end{array}\right)+\\ \qquad{{\mathcal{R}}}_{m,n}{u}_{m,n}+{u}_{m,n}^{3}-b{u}_{m,n}\end{array}$$

where **u** is a vector containing the values of the function *u*_*m*,*n*_ on the numerical grid, and (*dx*, *dy*) are the transverse steps. For each nonlinear equation, one finds the corresponding elements of the Jacobi matrix **J** through

6 $${J}_{(m,n),(p,q)}=\frac{\partial {f}_{m,n}({\bf{u}})}{\partial {u}_{p,q}}$$

The method consists in generating solution of corresponding system of nonlinear equations using the iterative procedure

7 $${{\bf{u}}}_{{\rm{new}}}={{\bf{u}}}_{{\rm{old}}}-{{\bf{J}}}^{-1}{\bf{f}}$$

where **f** is the vector with the elements given by Eq. (5). The iterations are stopped when the difference between solutions **u**_new_ and **u**_old_ reduces below the required level, typically below 10^−16^.

### The split-step Fourier method for solving the nonlinear Schrödinger equation

We rewrite Eq. (1) into

8 $$\frac{\partial \psi }{\partial z}={\mathcal{L}}\psi +{\mathcal{N}}\psi$$

with ${\mathcal{L}}=\frac{i}{2}({\partial }_{x}^{2}+{\partial }_{y}^{2})$ and ${\mathcal{N}}=i({\mathcal{R}}+| \psi {| }^{2})$ being linear diffraction and nonlinear operators, respectively. For small propagation steps one can treat/apply linear and nonlinear operators successively at each propagation step^82^. For instance, applying the Fourier transform to ${\mathcal{L}}\psi$ one obtains ${\mathcal{F}}\{{\mathcal{L}}\psi \}=-\frac{i}{2}({\omega }_{x}^{2}+{\omega }_{y}^{2})\hat{\psi }$, where $\hat{\psi }$ is the Fourier transform of *ψ*, *ω*_*x*,*y*_ are the frequencies. This allows to obtain complex field amplitude in Fourier domain on the next step *dz* as

9 $$\hat{\psi }(z+dz)={e}^{-\frac{i}{2}({\omega }_{x}^{2}+{\omega }_{y}^{2})dz}\hat{\psi }(z)$$

By taking inverse Fourier transform and applying the nonlinear operator one eventually obtains

10 $$\psi (z+dz)={e}^{{\mathcal{N}}dz}{{\mathcal{F}}}^{-1}\{{e}^{-\frac{i}{2}({\omega }_{x}^{2}+{\omega }_{y}^{2})dz}\hat{\psi }(z)\}$$

where ${{\mathcal{F}}}^{-1}$ is the inverse Fourier transform operator.

### Fs-laser inscription of the waveguide arrays

Fractal waveguide arrays were written in 10 cm long fused silica glass substrate (JGS1). The individual waveguides were inscribed by circularly polarized beam with central wavelength of 515 nm, with pulse duration of 230 fs, repetition rate 1 MHz, and pulse energy 270 nJ, focused with an aspheric lens (NA = 0.3) under the sample surface in the depth range from 600 to 1000 *μ*m near the preselected optimal depth of 800 *μ*m. Translation of the sample with respect to the focus was performed by a high-precision positioner (Aerotech) with a scanning velocity of 1 mm/s. Waveguides demonstrate propagation losses less than 0.3 dB/cm at *λ* = 800 nm. During writing process we keep the values of the distortion parameter within the range 0.2*a* < *r* < 0.8*a* to avoid overlap between neighboring elliptical waveguides. This guarantees the absence of uncontrollable distortions and excellent reproducibility of laser-written arrays.

### Experimental excitation of the waveguide arrays

In experiments, we employed single-waveguide excitations using fs pulses of variable energy *E* from 1 kHz Ti:sapphire laser at 800 nm central wavelength. Initially, short pulses with a 40 fs duration and wide spectrum from a regenerative amplifier system Spitfire HP (Spectra Physics) first pass through an active beam position stabilization system (Avesta) and an attenuator, and afterwards are launched into a 4*f* single-grating stretcher-compressor with a variable slit. Spectra of such pulses are narrowed by a slit down to 5 nm, which corresponds to the pulse duration of 280 fs. This increase in the pulse duration allows to prevent optical collapse and strong spectral broadening during pulse propagation in the waveguides, i.e. it allows to neglect the temporal effects. The pulses after stretcher compressor were focused into selected waveguides and the output intensity distributions after propagation in 10 cm sample were recorded using a Kiralux CMOS camera (Thorlabs). The input peak power in the waveguide (for each pulse in the 1 kHz sequence) was defined as a ratio of the input pulse energy *E* to the pulse duration *τ* = 280 fs. Taking into account the losses for the matching with the focusing lens the input power can be evaluated as 2.5 kW for each 1 nJ. For example, maximal excitation energy of *E* = 800 nJ in experimental patterns presented here corresponds to the peak power of 2.0 MW. Note that the waveguide array may be also manufactured in other materials^60,83^.

### Real-space polarization index

**Fig. 7:**
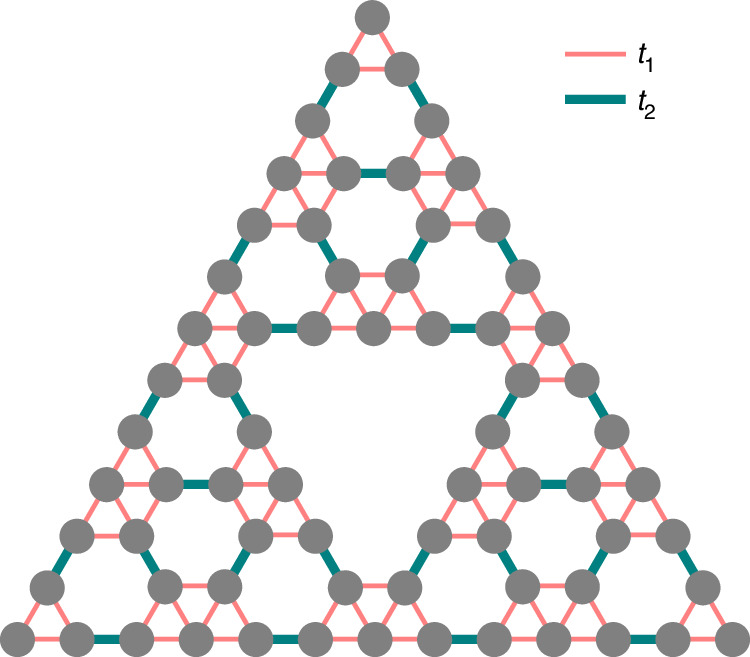
**Schematic illustration of third generation** *G*_3_ **of the Sierpiński gasket**. The intra-cell couplings between sites (gray circles) are indicated in this plot with thin red lines, while inter-cell couplings are indicated with thick blue lines. The intra-cell and inter-cell coupling strengths are given by *t*_1_ and *t*_2_, respectively

Fractal waveguide arrays are aperiodic structures that are sometimes considered as structures without bulk due to the method of their construction. In such structures the appearance of topological corner states can be associated with the nonzero real-space polarization index^36,62,63^, allowing to characterize topological properties of this system: ${P}=-{\frac{i}{2\pi}}\ln [\det (S)]$, where ${{S}_{m,n}}={Q}_{m}^{\dagger }{e}^{i2\pi \hat{q}/L}{Q}_{n}$, *L* is the length of the fractal array along the *q* direction, ${\hat{q}}$ is the position operator, *Q*_*n*_ is the eigenfunction of *n*^th^ state of the fractal array obtained with the periodic boundary conditions in the *q* direction (that is usually selected along the outer edge of the array) that lies below the corner state for which polarization index is calculated. Real-space polarization index for states in our system can be calculated using tight-binding approximation. In this approximation the fractal array, like *G*_3_ generation of the Sierpiński gasket, whose sites are depicted in Fig. 7, is described by the tight-binding Hamiltonian that accounts only for nearest-neighbor couplings of two types: the “intra-cell” coupling with coupling strength labeled as *t*_1_, and “inter-cell” coupling labeled as *t*_2_ (see Fig. 7, where these couplings are denoted by lines of different color). These coupling strengths are determined by the distortion parameter *r*, for *r* = 0.5*a* one has *t*_1_ = *t*_2_. To be able to apply periodic boundary conditions upon calculation of eigenfunctions *Q*_*n*_, we construct the rhombic structure from two stacked Sierpiński gasket arrays (shown in Fig. 7), real and virtual ones, neglecting coupling with the virtual and missing sites (see the image and description of corresponding structure in Supporting Information).

**Fig. 8:**
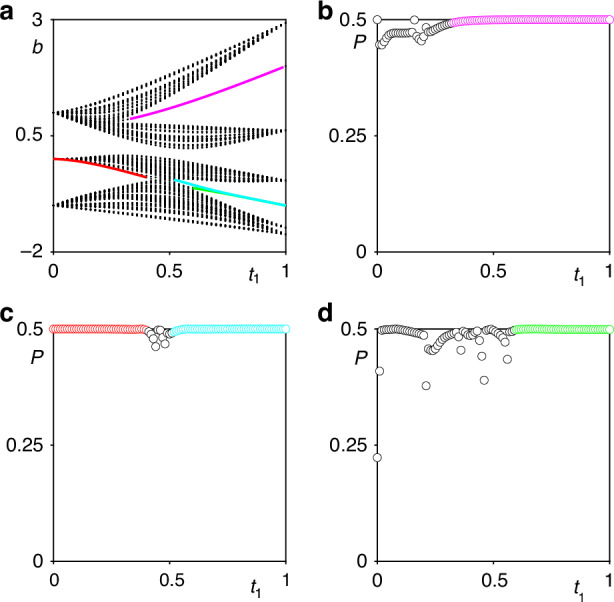
**Real-space polarization index**. **a** Spectrum of the *G*_4_ fractal lattice versus intra-cell coupling strength *t*_1_ obtained from tight-binding model. **b**–**d**) Real-space polarization index corresponding to the corner state indicated by magenta color, red and cyan colors, and green color in (**a**)

A detailed description of this approach and additional details can be found in Supporting Information, while here we show the results of the calculation. The linear spectrum of the fractal array obtained with the aid of the tight-binding approximation is shown in Fig. 8a. For consistency with the spectrum of Fig. 1b, obtained using a continuous model, we marked corner and all other localized states with the same colors as in Fig. 1b. As mentioned above, the calculation of the real-space polarization index for a given *r* requires knowledge of profiles of all eigenstates of the fractal array. In particular, for calculation of the real-space polarization index *P* for the magenta corner state laying in the gap of spectrum in Fig. 8a, one has to consider all eigenstates *Q*_*n*_ of array laying in the linear spectrum below this magenta curve, in accordance with formula for *P* provided above. The real-space polarization index for the magenta corner state is shown in Fig. 8b, and its value is 0.5 (i.e. quantized) exactly in the region where the corner state exists in the gap. This indicates on the topological nature of this state. However, magenta curve associated with corner state exists in the gap only within a finite range of distortion coefficients *r* in Fig. 1b (intra-cell coupling constants *t*_1_ in Fig. 8a). Outside this range the gap closes, corner state delocalizes and transforms into extended state in the band. The calculation of *P* for such extended eigenstate with the same index *n* as for the magenta curve yields rapidly changing with *t*_1_ non-quantized value of *P*. It is nonzero only because the state *n*, for which calculation is performed is taken in the depth of the band. Similar results are obtained for other corner state branches. Red and cyan branches from Fig. 8a formally belong to the same curve that exist in different parameter regions, so one can calculate the real-space polarization index *P* assuming that the bands below this curve are filled. The calculated *P* value is indeed 0.5 in two parameter regions that correspond to the red and cyan curves laying within corresponding gaps, as shown in Fig. 8c. Finally, the calculation of polarization index for the green corner state again demonstrates that this state is topologically nontrivial, see Fig. 8d. Just as in the case of a magenta branch, for all other corner states real-space polarization index is quantized in the gap, but becomes non-quantized if calculation is continued in the band for the extended eigenstate with the same index *n* as gap eigenstate. Thus, case-2 fractal structure can support topologically nontrivial modes in both domains *t*_1_ > 0.5 and *t*_1_ < 0.5 (i.e., domains *r* < 0.5*a* and *r* > 0.5*a* in Fig. 1). Note that if all the bands in Fig. 8 are filled (i.e. if the index is calculated for the extended state that has largest propagation constant among all modes), the real-space polarization is zero throughout the region 0 < *t*_1_ < 1.

## Supplementary information

Supplementary Information for Observation of nonlinear fractal higher-order topological insulator
