# Supplementary material for: Observation of nonlinear fractal higher order topological insulator
Source: Light Sci Appl. 2024 Sep 20;13:264. doi: 10.1038/s41377-024-01611-1 (PMC11413017; doi:10.1038/s41377-024-01611-1)
Supplement: Supplementary file 1 — Supplementary Information for Observation of nonlinear fractal higher-order topological insulator [file 41377_2024_1611_MOESM1_ESM.pdf]

Supplementary Information *for* Observation of nonlinear fractal higher-order topological insulator

Hua Zhong,[1,*] Victor O. Kompanets,[2,*] Yiqi Zhang,[1,†] Yaroslav V. Kartashov,[2,‡] Meng Cao,[1] Yongdong Li,[1] Sergei A. Zhuravitskii,[2,3] Nikolay N. Skryabin,[2,3] Ivan V. Dyakonov,[3] Alexander A. Kalinkin,[2,3] Sergei P. Kulik,[3] Sergey V. Chekalin,[2] and Victor N. Zadkov[2,4]

[1]*Key Laboratory for Physical Electronics and Devices, Ministry of Education, School of Electronic Science and Engineering, Xi'an Jiaotong University, Xi'an 710049, China*
[2]*Institute of Spectroscopy, Russian Academy of Sciences, Troitsk, Moscow, 108840, Russia*
[3]*Quantum Technology Centre, Faculty of Physics, M. V. Lomonosov Moscow State University, Moscow, 119991, Russia*
[4]*Faculty of Physics, Higher School of Economics, Moscow, 105066, Russia*

I. TWO SIERPIŃSKI GASKET STRUCTURES WITH THE SAME FIRST GENERATION

In this work, we use the same first-generation waveguide array to construct two different types of Sierpiński gasket structures. They are further referred to as case-1 and case-2 structures, and the difference in their construction is outlined in Fig. S1. While case-1 structure is obtained by combining the previous generations without overlap of their waveguides, in more compact case-2 structure vertices of the previous-generation triangular structures overlap (as indicated by the green dots in Fig. S1) leading to more compact array (compare G_n structures with $n > 1$ in the top and bottom rows of Fig. S1). Even though nonlinear excitations in case-1 Sierpiński gasket arrays were studied before theoretically, their experimental observation was not reported to the date. While main text illustrates the results for the case-2 arrays, here, for completeness, we present experimental observation of the corner solitons in the case-1 fractal structure.

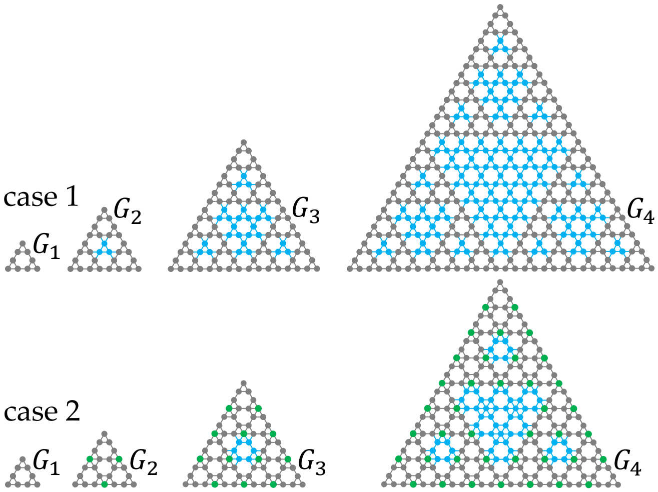

FIG. S1. Iterative generation of the Sierpiński gasket structures of two different types. The first generation G_1 of the Sierpiński gasket has 9 sites, and the nth generation G_n includes three copies of G_{n-1}. In the case-1 structure sites, in vertices of previous generations do not overlap, while in the case-2 structure they overlap, as shown by the green dots in the second row. Consequently, there are 3^{n+1} sites in case-1 G_n array and there are $3^{n+1} - (3^n - 3)/2$ sites in case-2 G_n array. In this figure gray dots show sites of the constructed fractal structure, green dots in the second row highlight the sites that overlap upon construction of the next-generation structures, while cyan dots show "missing" sites in the final fractal structure in comparison with the structure with the periodic bulk.

II. THE SCHEME OF THE CHARACTERIZATION SETUP

For experimental observation of topological corner solitons in fractal HOTIs we use characterization setup schematically depicted in Fig. S2. Radiation at 800 nm central wavelength from 1 kHz Ti:sapphire laser system Spitfire HP (Spectra Physics) enters into a single-grating $4f$ pulse slicer, that cuts 5 nm-wide portion from the initial 40 nm-wide spectrum. The duration of pulses after slicer amounts to 280 fs (FWHM). Passage of radiation through the pulse slicer is required to prevent collapse during propagation in waveguides of initial 40 fs pulses delivered by laser system. Next, the pulses pass through an active beam stabilization system (Avesta) and are focused by an optically matched achromatic lens onto the sample. The pulse energy is varied by a 2 mm-thick quartz continuously variable neutral density filter and is controlled by a power meter. The output intensity distributions from the waveguide arrays are recorded by a scientific CMOS camera Kiralux (Thorlabs) with an 8X microscope objective lens. The input peak power in the waveguide defined as the ratio of the pulse energy E to the pulse duration t can be evaluated as 2.5 kW for each 1 nJ.

* The two authors constribute equally to this work.
† zhangyiqi@xjtu.edu.cn
‡ yaroslav.kartashov@icfo.eu

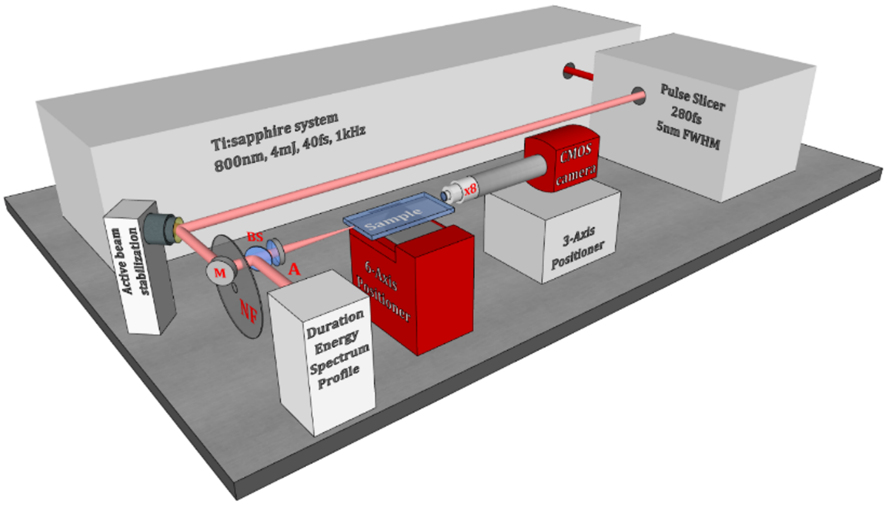

FIG. S2. Characterization setup. NF – Continuously variable ND filter, BS – Ultrafast beam splitter, A – Achromatic lens.

III. LINEAR STABILITY ANALYSIS

To perform linear stability analysis of the obtained topological corner solitons, we write perturbed solutions in the form

$$\psi = \left[u(x,y) + v(x,y)e^{\beta z} + w^*(x,y)e^{\beta^* z}\right]e^{ibz} \qquad \text{(S1)}$$

where $v, w \ll u$ are small perturbations, u is the function describing soliton profile, and $\beta = \beta_{\rm re} + i\beta_{\rm im}$ is the perturbation growth rate that can be complex. Inserting Eq. (S1) into Eq. (1) in the main text and linearizing it around stationary solution u, we arrive at the linear eigenvalue problem:

$$i\beta v = -\frac{1}{2}\left(\frac{\partial}{\partial x^2} + \frac{\partial}{\partial y^2}\right)v - (\mathcal{R} - b)v - 2|u|^2 v - |u|^2 w$$
$$i\beta w = +\frac{1}{2}\left(\frac{\partial}{\partial x^2} + \frac{\partial}{\partial y^2}\right)w + (\mathcal{R} - b)w + 2|u|^2 w + |u|^2 v \qquad \text{(S2)}$$

Solving the problem in Eq. (S2) using standard eigenvalue solver, we obtain the dependence of the perturbation growth rate β (and associated perturbation profiles v, w) on the propagation constant b for a given family of nonlinear states. If real part of the perturbation growth rate $\beta_{\rm re} \le 0$ for all possible perturbations, corresponding nonlinear state u is linearly stable and in the presence of small perturbations it will exhibit only small-amplitude oscillations upon evolution. Otherwise, if at least one perturbation mode has $\beta_{\rm re} > 0$, the nonlinear state is unstable and will decay in the course of propagation.

Such an analysis was performed for all soliton families for case-2 configuration that are presented in Fig. 2 of the main text. It was found that only one green family from Fig. 2**b** can be partially unstable, while for all other corner soliton families $\beta_{\rm re} \le 0$ that indicates on their stability. The dependence $\beta_{\rm re}(b)$ for green family from Fig. 2**b** is presented in Fig. S3. Perturbation growth rate becomes nonzero exactly for the dashed segment of the green line in Fig. 2**b**, i.e. the results of linear stability analysis fully confirm the results of direct propagation of perturbed solitons. They also clearly indicate that soliton solutions may remain stable even when they enter into spectral bands and couple with bulk modes.

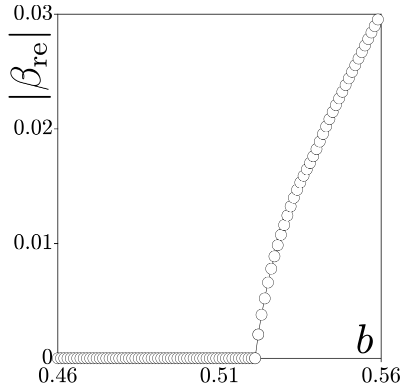

FIG. S3. Maximal real part of the perturbation growth rate $\beta_{\rm re}$ versus propagation constant b for the nonlinear corner state family corresponding to the green curve in Fig. 2**b** in the main text.

IV. REAL-SPACE POLARIZATION INDEX

Fractal waveguide arrays are aperiodic, but regular structures that posses multiple internal holes, corners, and edges. On this reason, such topological indices as Chern and winding numbers are not applicable for characterization of topological properties of fractal systems. Topological transitions in such systems can be instead characterized by the real-space polarization index [1, 2]. Since real-space polarization index for fractal HOTI

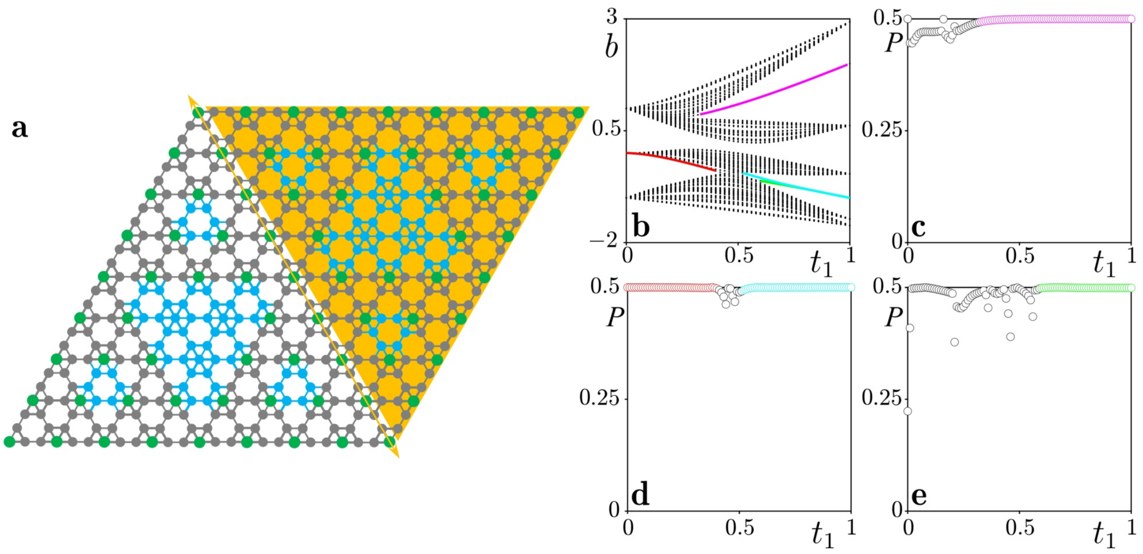

FIG. S4. (**a**) Rhombic fractal structure composed from two case-2 Sierpiński gaskets. (**b**) Propagation constants b of eigenmodes of the structure from panel (**a**) versus intra-cell coupling strength t_1 obtained from tight-binding model. (**c**) Real-space polarization corresponding to the corner state indicated by magenta color in (**b**). (**d**) Real-space polarization corresponding to the corner state indicated by red and cyan colors in (**b**). (**e**) Real-space polarization corresponding to the corner state indicated by green color in (**b**).

based on case-1 Sierpiński gasket was discussed in previous theoretical work [3], here we show the results only for the case-2 Sierpiński gasket. To introduce real-space polarization, we adopt fractal rhombic structure composed from two case-2 Sierpiński gaskets (we use the G_4 generation as an example), which have the common "edge" indicated by the double-sided yellow arrow in Fig. S4**a**. We enumerate all sites in this structure continuously, but set to zero the coupling with "missing" cyan sites and sites with yellow background. Using tight-binding approximation, one can directly construct the Hamiltonian of this composite case-2 Sierpiński gasket structure, with intra-cell and inter-cell coupling strengths being t_1 and $t_2 = 1 - t_1$, respectively (it should be pointed out that green sites in Fig. S4**a** can be considered as cell centers surrounded by six nearest-neighbor sites, when introducing intra- and inter-cell coupling strengths t_1 and t_2). The eigenvalues of the so-constructed tight-binding Hamiltonian H (after removal of meaningless zero eigenvalues) are presented in Fig. S4**b** as functions of t_1. For consistency with spectrum of Fig. 1**b** in the main text (calculated in the frames of continuous model taking into account all details of the refractive index distribution, where coupling strengths are controlled by the waveguide shift r), in Fig. S4**b** we marked corner and all other localized states with the same colors as in Fig. 1**b**. It should be mentioned that corner states marked with green and cyan colors obtained in the frames of the tight-binding model may slightly differ from states obtained in continuous model due to elliptical shape of waveguides, that breaks $\mathcal{C}_3$ discrete rotational symmetry of case-2 fractal structure assumed in idealized tight-binding model.

Real-space polarization index for fractal structure considered here can be written as

$$P = -\frac{i}{2\pi}\ln[\det(S)] \qquad \text{(S3)}$$

where

$$S_{m,n} = Q_m^{\dagger} e^{i2\pi\hat{q}/L} Q_n \qquad \text{(S4)}$$

L is the length of the array along q direction, $\hat{q}$ is the position operator (sites in one unit cell have the same coordinates), Q_n is the eigenfunction of nth state of the fractal array obtained with periodic boundary conditions in the q direction. Notice that the direction q is parallel either to the horizontal edge of rhombic array from Fig. S4**a** or to its left inclined edge. For structure presented in Fig. S4**a** one has $L = 9$. When calculating real-space polarization index corresponding, for example, to the magenta corner state in the gap in Fig. S4**b**, one has to assume that the bands below magenta curve are filled. This gives quantized real-space polarization index $P = 0.5$ (see red hollow dots in Fig. S4**c**) in the region where magenta curve exists in the gap (notice that this curve is encountered not only at $t_1 > 0.5$, but it also extends into parameter domain where $t_1 < 0.5$ that is very similar to behavior of this curve in continuous model). However, magenta curve associated with corner state exists in the gap only within finite range of the intra-cell coupling strength t_1. Outside this range the gap closes, corner state delocalizes and transforms into extended state in the band. We still perform the calculation of P for the extended eigenstate with the same index n as for magenta curve, but actually this is somewhat meaningless procedure (due to closure of corresponding gap) for state in the depth of

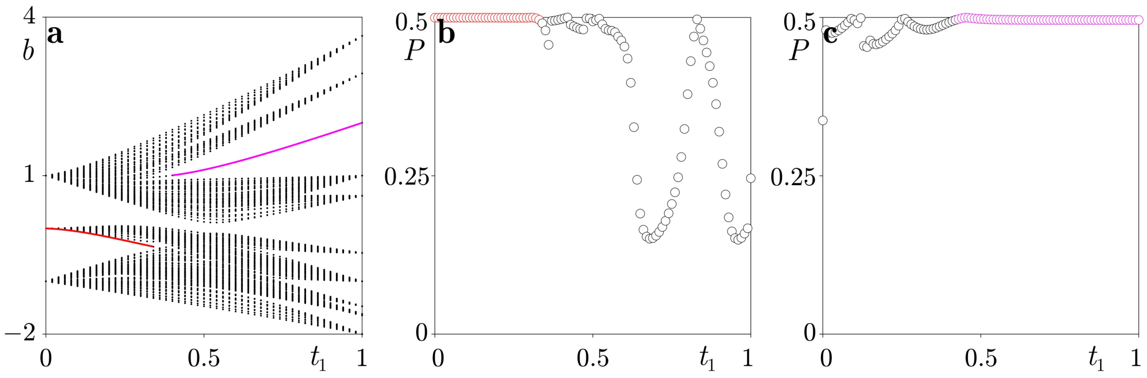

FIG. S5. (**a**) Propagation constants b of eigenmodes of the nonfractal structure versus intra-cell coupling strength t_1 obtained from tight-binding model. (**b**) Real-space polarization corresponding to the corner state indicated by red color in (**a**). (**c**) Real-space polarization corresponding to the corner state indicated by magenta colors in (**a**).

the band. On this reason we obtain in this last regime rapidly changing with t_1 non-quantized value of P, as shown by the black hollow dots in Fig. S4**c**. It is nonzero only because the state n, for which calculation is performed is taken in the depth of the band. Similar results are obtained for all other topological corner states (including red and cyan branches in Fig. S4**b**) — real-space polarization index Fig. S4**d** is quantized for corner states in the gap, but becomes non-quantized if calculation is continued in the band for the extended eigenstate with the same index as gap eigenstate. Finally, calculation of real-space polarization index for green corner state again demonstrates that this state is topologically nontrivial, see Fig. S4**e**. Therefore, case-2 fractal structure can support topologically nontrivial modes in both domains, at $t_1 > 0.5$ and at $t_1 < 0.5$.

To calculate real-space polarization index for similar, but non-fractal lattice, we still use the configuration shown in Fig. S4**a**, where we set to zero the coupling with sites with yellow background, but now we do take into account the coupling with cyan sites that was omitted in fractal case. The spectrum of the non-fractal lattice is shown in Fig. S5**a**. One finds that the corner states indicated by red and magenta curves still exist in linear spectrum, but those that were shown by green and cyan curves in fractal structure actually disappear (merge with the band) in this tight-binding model (as shown in the main text, in continuous system they still can appear, but for large shifts r). Therefore, the difference between the fractal and non-fractal structures is more pronounced in the tight-binding method that is utilized here for topological characterization of the system. The real-space polarization index corresponding to the red and magenta curves in spectrum of non-fractal system is displayed in Figs. S5**b** and S5**c**, respectively, and corresponding values are indeed 0.5 in the region where corner states appear in the gap, indicating on the fact that such states are topological. By comparing the results shown in Figs. S5**b** and S4**d** and the results in Figs. S5**c** and S4**c**, one can conclude that the existence domains for corner states corresponding to red and magenta curves are somewhat larger in fractal structure. This confirms that in a fractal structure the range of existence of certain topological corner states can be extended in comparison with a non-fractal one.

V. SOLITON FAMILIES IN G_4 STRUCTURES

To confirm that the properties of solitons remain qualitatively similar in fractals of different generations, here we present soliton families and representative field modulus distributions in G_4 fractal arrays, see Fig. S6. These soliton families are also displayed in the main text, see Figs. 2**i** and 2**j**. Clearly, all the nonlinear families obtained in G_3 structure also exist in G_4 arrays, while corresponding soliton solutions demonstrate similar shape transformations, when their propagation constants increase and enter into allowed bands. Notice that all solitons corresponding to dots 1, 3, 7, 9 and 11 are clearly localized in the gap where they bifurcate from corresponding topological modes. Example of delocalized nonlinear mode (orange branch, modes 5 and 6) is presented too.

VI. COMPARISON OF PROPAGATION IN FRACTAL AND NON-FRACTAL STRUCTURES

To further compare fractal and non-fractal structures, we show the results of dynamical excitation (in linear case) of the same sites in G_4 fractal structure and its non-fractal counterpart. Here, we deliberately selected larger G_4 structure to illustrate that propagation dynamics in it is similar to dynamics in G_3 arrays considered in the main text. In addition, we took larger propagation distance of $z = 300$ (in comparison with sample length corresponding to $z = 88$) to clearly illustrate that localization features (in the cases when topological modes are excited) are observed at any distances. The output intensity distributions in G_4 fractal and non-fractal struc-

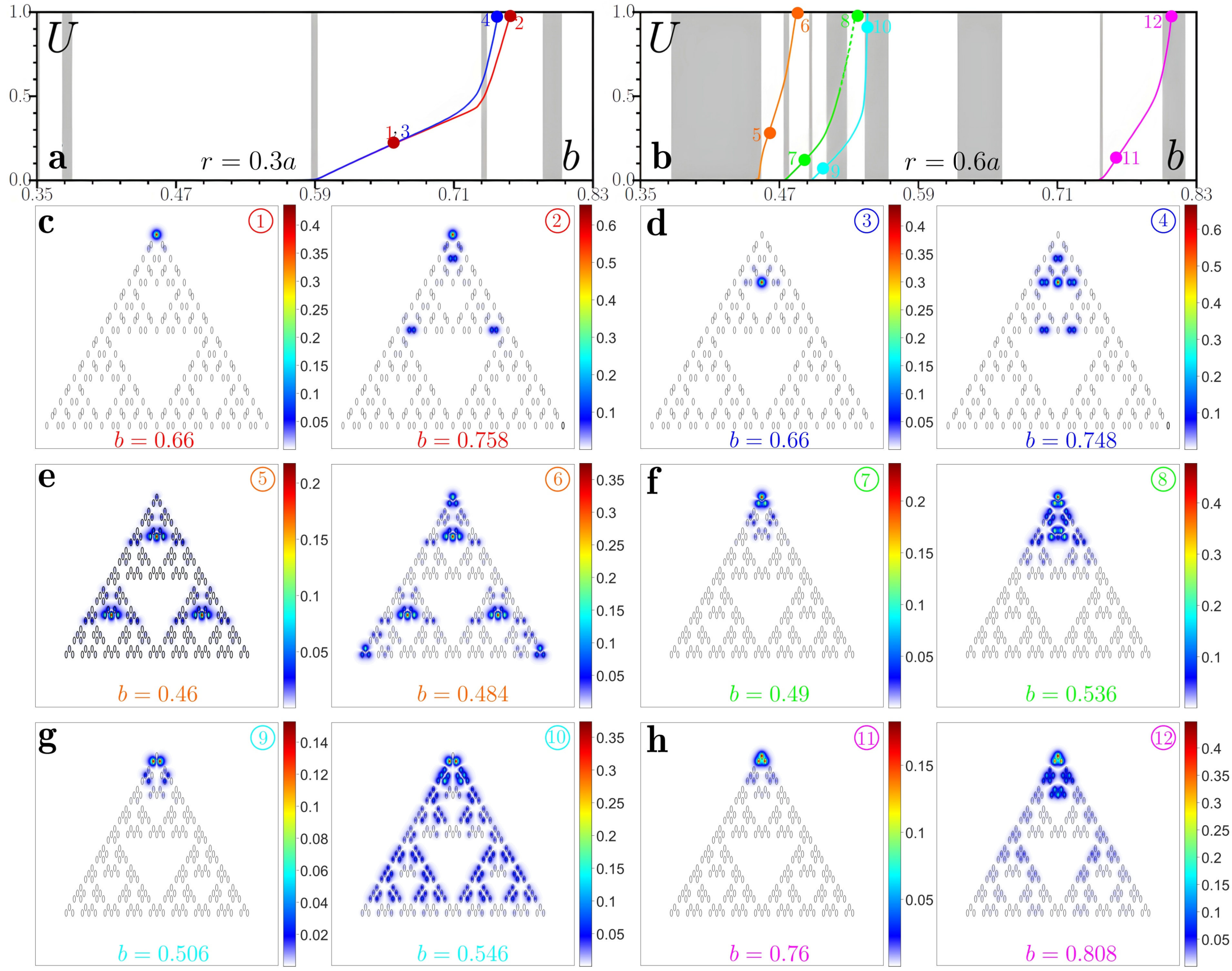

FIG. S6. Topological solitons in G_4 fractal insulators. (**a**) Power vs propagation constant for solitons bifurcating from the linear eigenstates concentrated around sites 1 (red curve) and 4 (blue curve) in the fractal array with $r = 0.3a$. Panels (**c**) and (**d**) show field modulus distributions corresponding to the dots in (**a**). (**b**) Power vs propagation constant for the solitons bifurcating from linear eigenstates concentrated around sites 1 (green, cyan and magenta curves) and 4 (orange curve) in fractal array with $r = 0.6a$. Field modulus distributions corresponding to the dots in (**b**) are shown in (**e**)–(**h**). In (**a**) and (**b**) gray regions are associated either with the eigenvalues of the localized states or with the bands of the delocalized states.

tures are presented in Fig. S7. Notice that the output intensity distributions in fractal and non-fractal structures are rather similar at $r < 0.5a$, as shown in the left two columns of Fig. S7. This is natural for corner states in the outer corners that are topological in both structures. The excitation in the bulk of non-fractal array depicted in Fig. S7**j** also shows surprisingly good localization. The output patterns differ strongly at $r = 0.5a$, see two central columns in Fig. S7, as typically the expansion is larger in non-fractal structure. At $r > 0.5a$, see two right columns in Fig. S7, the excitation of corner waveguide leads to good localization in both fractal and non-fractal geometries. At the same time, Fig. S7**l** illustrates the example, where dynamics in fractal and non-fractal arrays can be substantially different.

VII. CASE-1 SIERPIŃSKI GASKET ARRAYS

Schematic representations of the case-1 Sierpiński gasket arrays of the third generation G_3 with $r = 0.3a$ and $r = 0.5a$ (here r is the parameter that describes the waveguide shift, as indicated schematically in the bottom panel) are presented in Fig. S8**a**. In the figure the blue sectors highlight (i.e. coincide with) first-generation structures G_1, while orange sectors highlight second-generation structures G_2. Case-1 Sierpiński gasket waveguide array from Fig. S8**a** can also realize fractal higher-order topological insulator (HOTI). To illustrate when this structure enters into topological phase, in Fig. S8**b** we show the linear spectrum of third-generation array G_3 as a function of shift parameter r. A surprising result is that the corner states that are highlighted

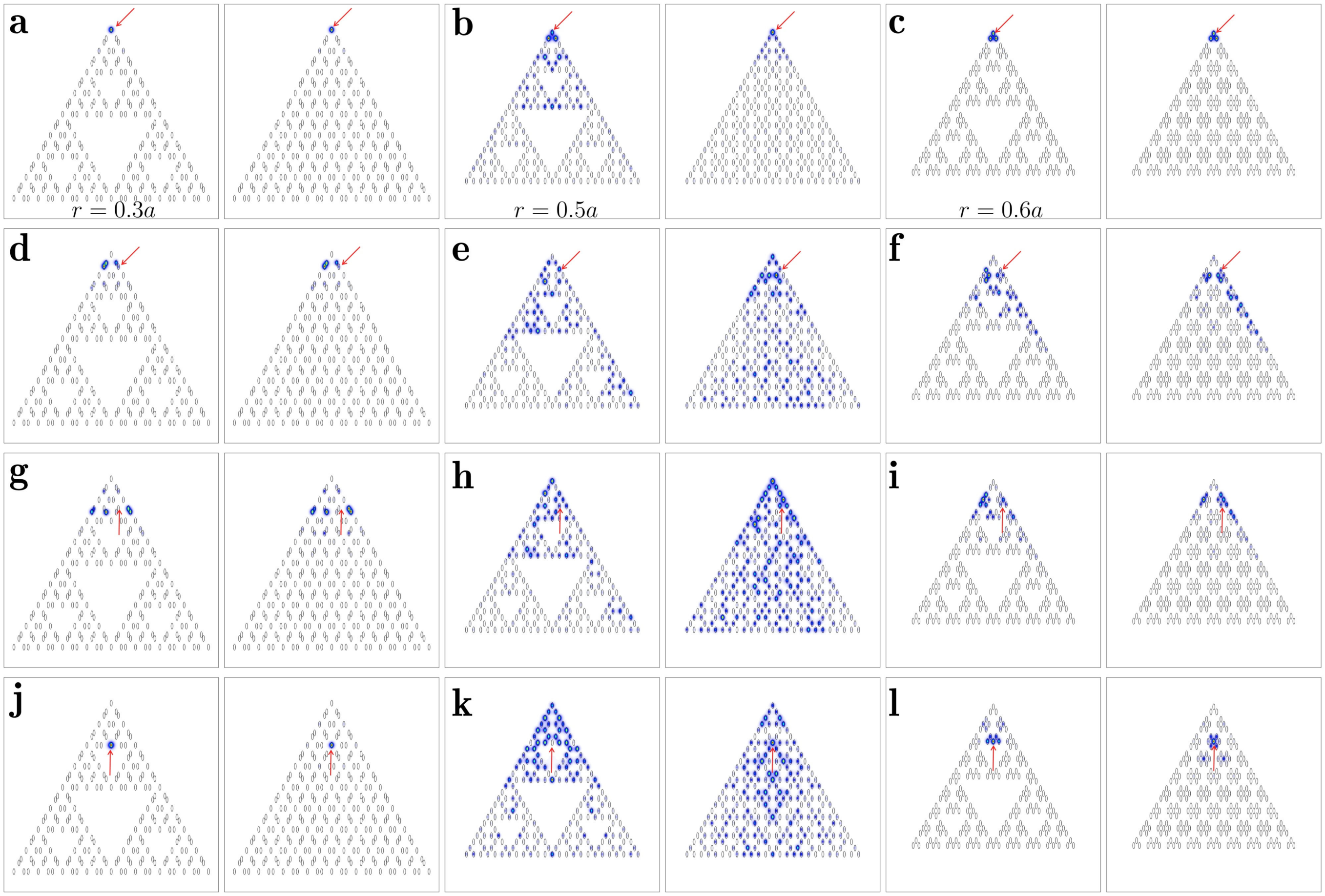

FIG. S7. Output intensity distributions for single-site excitations indicated by the red arrow after a propagation distance of $z = 300$ in both fractal and non-fractal structures. Left two columns (**a**, **d**, **g**, **j**) are for the cases with $r = 0.3a$, middle two columns (**b**, **e**, **h**, **k**) are for the cases with $r = 0.5a$, and right two columns (**c**, **f**, **i**, **l**) are for the cases with $r = 0.6a$. The array profiles are indicated by ellipses.

in this spectrum by the red curve exist in a wide range of r values, beyond the $r < 0.5a$ region, where the corner states are usually encountered in non-fractal HOTIs constructed on periodic arrays. The range of existence of the corner states in case-1 structure is also substantially broader than that in previously reported realizations of the fractal HOTIs on Sierpiński carpet arrays. Notice that at $r > 0.5a$ corner states exist in a very narrow gap in the spectrum that is not visible on the scale of Fig. S8**b**. Representative intensity distributions of linear corner states corresponding to the dots 1 and 2 are presented in Fig. S8**c**. The family of corner solitons bifurcating from linear corner states at $r = 0.3a$ is shown in Fig. S8**d**. Notice strong localization of corner soliton in the gap lying below edge state band (see intensity distribution in state labeled 3 in Fig. S8**e**), and the possibility of nonlinearity-induced coupling of corner soliton with edge states from internal edge (see state labeled 4 in Fig. S8**e**). For even higher powers U this corner state enters into the band of delocalized states.

VIII. OBSERVATION OF NONLINEAR CORNER STATES IN CASE-1 FRACTAL HOTI

Case-1 Sierpiński gasket waveguide arrays were inscribed in fused silica samples using fs-laser writing technique described in the main text. Similar to the case-2 structures, in the case-1 Sierpiński gasket arrays we excited four different representative waveguides indicated by arrows with numbers in Fig. S8**a**. The comparison of experimental and theoretical results for the fractal array with $r = 0.3a$ is presented in Fig. S9. When corner site 1 is excited, as shown in Fig. S9**a**, one observes the formation of corner soliton at all employed pulse energies (power levels in theory). This clearly confirms the existence of linear and bifurcating from them thresholdless nonlinear corner states in case-1 fractal HOTI. In contrast, when sites 2, 3, or 4 are excited, which are located on the outer or inner edges of fractal structure (site 4 also coincides with the vertex of G_1 generation structure), one observes diffraction at the lowest pulse energies (see output intensity distributions at $E = 10\,\text{nJ}$

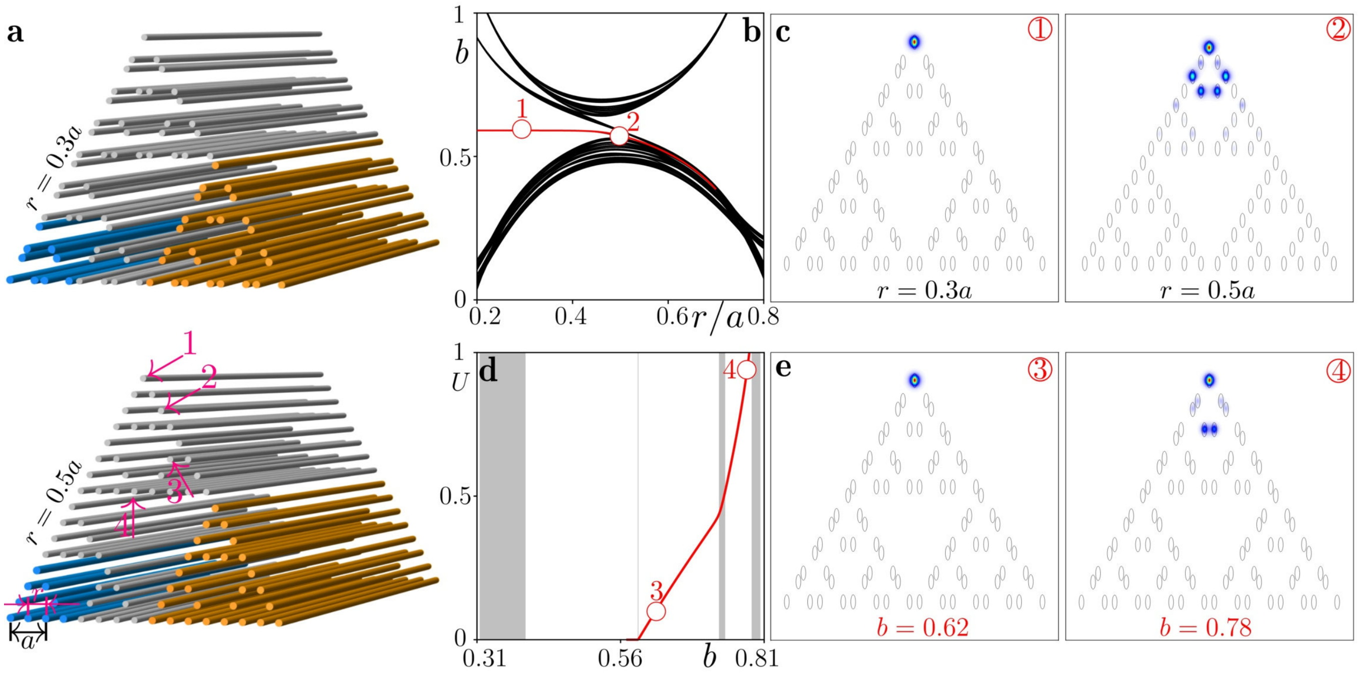

FIG. S8. (**a**) The case-1 Sierpinski gasket waveguide arrays with different shift parameter r. (**b**) Dependence of propagation constants b of linear eigenmodes of case-1 array on the shift parameter r. Black curves correspond to extended states, while red curve corresponds to corner states. (**c**) Representative intensity distributions in corner states corresponding to the dots in (**b**). (**d**) Nonlinear corner state family bifurcating from the linear corner state at $r = 0.3a$. Gray regions correspond to the bands of the extended states, gray line corresponds to the eigenvalue of the linear corner state. (**e**) Intensity distributions of nonlinear corner states corresponding to the dots in (**d**). In all cases the array depth is $p = 5.7$.

in Figs. S9**b**-S9**d**). Increasing pulse energy leads to arrest of diffraction to some extent, but even at the highest energies $E = 800\,\mathrm{nJ}$ one typically observes beatings of power between a pair of closely located waveguides, without clear evidence of single-site soliton formation. It should be mentioned that in comparison with case-2 HOTIs described in the main text, for selected value of $r = 0.3a$ in case-1 HOTI there are fewer opportunities for excitation of well-localized topological solitons, since in the latter structures they can only appear in the outer corners, while in the former structures well-localized nonlinear states may form in each inner corner (the number of such corners increases with increase of the order of array generation). These experimental observations fully agree with theoretical output intensity distributions (see plots with white background).

We also investigated nonlinear excitations in case-1 fractal Sierpiński gasket array with $r = 0.5a$, as shown in Fig. S10. In this case, linear corner state exists too, but it is much less localized than at $r = 0.3a$ and it extends over several sites of the structure (see the mode numbered 2 in Fig. S8**c**). On this reason, its excitation efficiency with single-site beam in linear regime $E = 10\,\mathrm{nJ}$ is rather low and light at the output considerably spreads over the array (Fig. S10**a**). With increase of input pulse energy the transition to localization for site 1 is observed at $E \sim 450\,\mathrm{nJ}$ (see the panels with $E = 450\,\mathrm{nJ}$ and $E = 800\,\mathrm{nJ}$ in Fig. S10**a**). For excitation of all other sites 2, 3, or 4, one observes strong diffraction in the linear regime and transition to localization at larger pulse energies $E \sim 700\,\mathrm{nJ}$, see Figs. S10**b**-S10**d**. Thus, one can conclude that in the regime $r \geq 0.5a$ the existence of moderately localized corner states simplifies dynamical excitation with single-site beams, but in experiments this is manifested only in somewhat reduced energy level for the onset of localization in corner site.

Comparing the results for case-2 Sierpiński gasket array described in the main text, and case-1 array described here, one can notice somewhat richer opportunities for control of localization degree, position, and waveforms of localized excitations in the former HOTIs, opening interesting prospects for control of light propagation regimes and paths in such structures.

[1] Wheeler, W. A., Wagner, L. K. & Hughes, T. L. Many-body electric multipole operators in extended systems. *Phys. Rev. B* **100**, 245135 (2019).

[2] Kang, B., Shiozaki, K. & Cho, G. Y. Many-body order

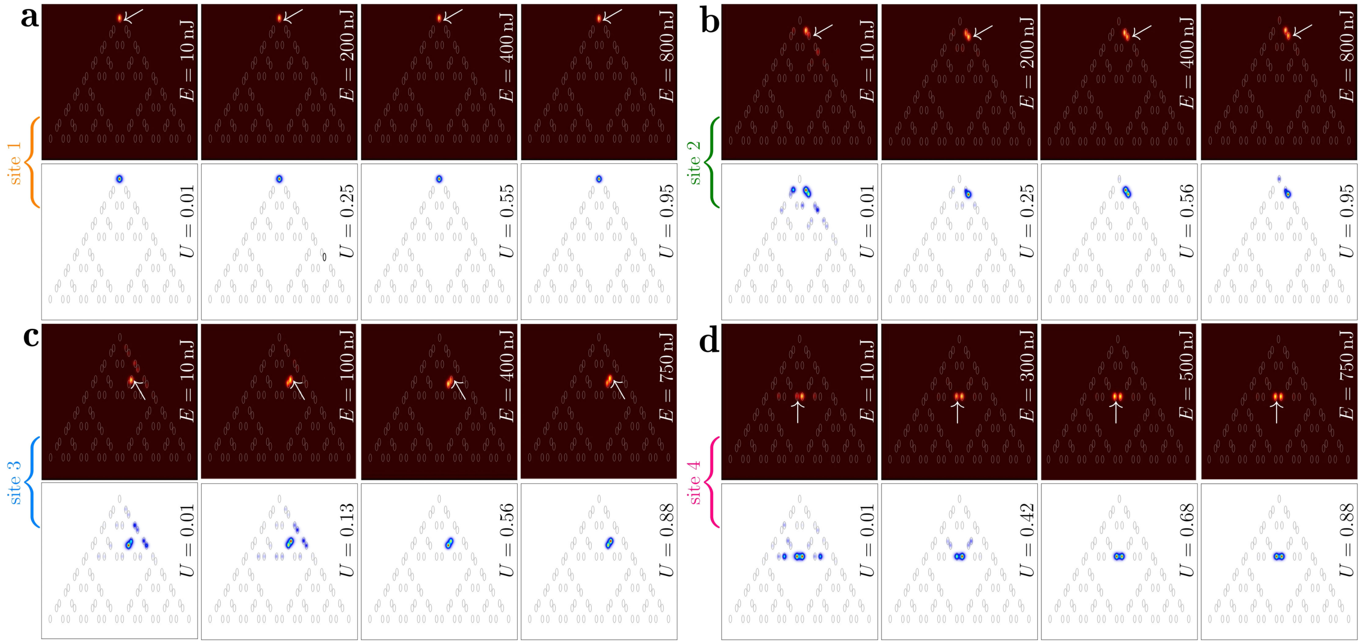

FIG. S9. Observation of nonlinear states in fractal array with $r = 0.3a$. Output intensity distributions after 10 cm of propagation for excitation of sites 1 (**a**), 2 (**b**), 3 (**c**) and 4 (**d**) in G_3 case-1 fractal array. Figures with maroon background show experimental results, while figures with white background show results of the theoretical simulations. Gray ellipses in all panels indicate waveguide locations. Input pulse energies E and powers U are indicated on each plot. All distributions are shown within the window $-26 \leq x, y \leq 26$. Arrows in the experimental panels indicate position of the input excitation.

parameters for multipoles in solids. *Phys. Rev. B* **100**, 245134 (2019).

[3] Ren, B. *et al.* Theory of nonlinear corner states in photonic fractal lattices. *Nanophoton.* **12**, 3829–3838 (2023).

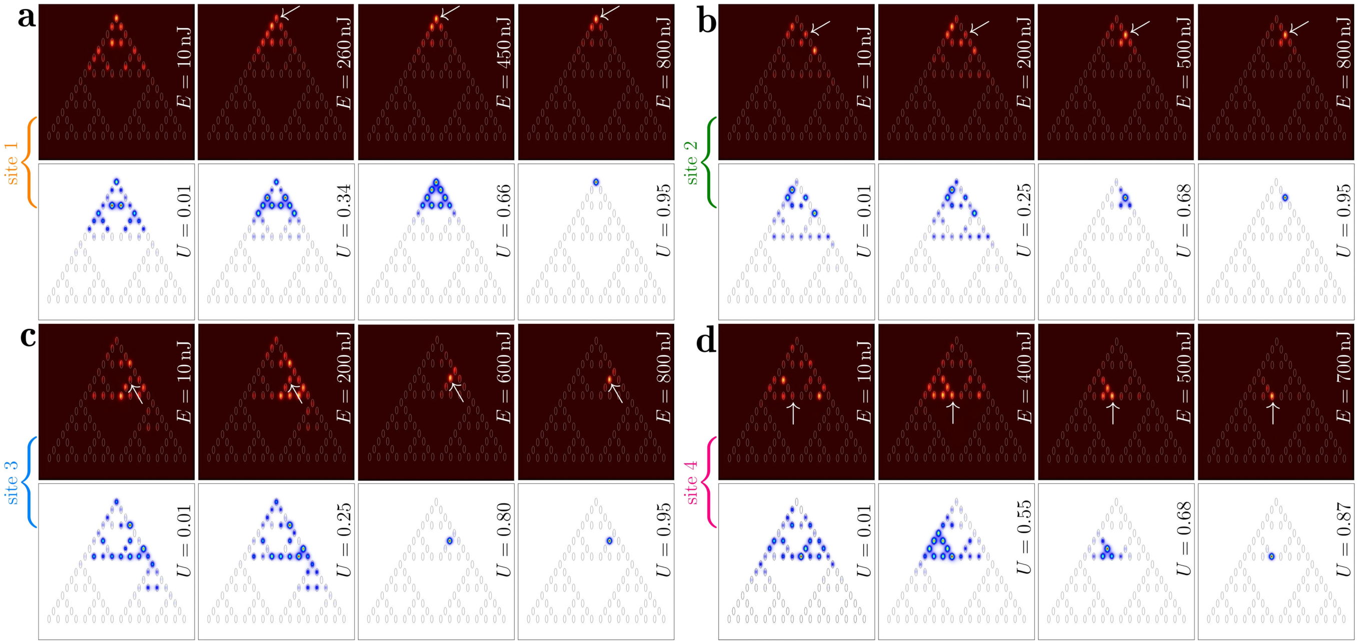

FIG. S10. Nonlinear dynamics in fractal case-1 G_3 array with $r = 0.5a$. For excitation of all four sites 1 (**a**), 2 (**b**), 3 (**c**) and 4 (**d**) one observes rich nonlinear dynamics with considerable expansion of the pattern at low powers and contraction to practically single site at the highest powers. Arrows in experimental panels indicate position of the input excitation.
